# Inefficient exploitation of accessory receptors reduces the sensitivity of chimeric antigen receptors

**DOI:** 10.1073/pnas.2216352120

**Published:** 2023-01-04

**Authors:** Jake Burton, Jesús A. Siller-Farfán, Johannes Pettmann, Benjamin Salzer, Mikhail Kutuzov, P. Anton van der Merwe, Omer Dushek

**Affiliations:** ^a^Sir William Dunn School of Pathology, University of Oxford, OX1 3RE Oxford, UK

**Keywords:** T cells, chimeric antigen receptors, adhesion receptors, antigen sensitivity

## Abstract

Chimeric antigen receptors (CARs) can redirect T cells to target abnormal cells, but their activity is limited by a profound defect in antigen sensitivity, the source of which remains unclear. Here, we show that CARs have a > 100-fold lower antigen sensitivity compared to the T cell receptor (TCR) when antigen is presented on antigen-presenting cells (APCs) but nearly identical sensitivity when antigen is presented as purified protein. We next systematically measured the impact of engaging important T cell accessory receptors (CD2, LFA-1, CD28, CD27, and 4-1BB) on antigen sensitivity by adding their purified ligands. Unexpectedly, we found that engaging CD2 or LFA-1 improved the antigen sensitivity of the TCR by 125- and 22-fold, respectively, but improved CAR sensitivity by only < 5-fold. This differential effect of CD2 and LFA-1 engagement on the TCR vs. CAR was confirmed using APCs. We found that sensitivity to antigen can be partially restored by fusing the CAR variable domains to the TCR CD3*ε* subunit (also known as a TRuC) and fully restored by exchanging the TCR*αβ* variable domains for those of the CAR (also known as STAR or HIT). Importantly, these improvements in TRuC and STAR/HIT sensitivity can be predicted by their enhanced ability to exploit CD2 and LFA-1. These findings demonstrate that the CAR sensitivity defect is a result of their inefficient exploitation of accessory receptors and suggest approaches to increase sensitivity.

## Significance

T cells are engineered to recognize cancer antigens using chimeric antigen receptors (CARs). This therapy is approved to treat B cell cancers, but patients relapse with B cells that express low levels of the antigen. It is now clear that CARs have a profound defect in antigen sensitivity. They require > 100-fold more antigen to activate T cells compared to their native T cell antigen receptors (TCRs). Here we demonstrate that the antigen sensitivity defect of CARs is a result of their inability to fully exploit adhesion receptors. Modifying CARs so that they more closely resemble the TCR allows them to efficiently exploit adhesion receptors and fully restores their antigen sensitivity. The work suggests to improve CAR-T cell therapy.

Adoptive cell transfer (ACT) of genetically engineered T cells expressing chimeric antigen receptors (CARs) is a clinically approved cancer therapy for hematological malignancies (1, 2). CARs are synthetic receptors that are typically generated by the fusion of an antibody-derived, antigen-binding single-chain variable fragment (scFv) with intracellular signaling motifs from the cytoplasmic tails of the T cell receptor (TCR) complex. Although administration of CAR-T cells targeting the surface antigens CD19, CD20, and B cell maturation antigen (BCMA or CD269) on malignant B cells results in an excellent initial response, patients often relapse when malignant cells emerge with reduced levels of target antigens (3–8). One likely explanation for this escape is that CARs require 100- to 1000-fold higher antigen densities to induce T cell activation compared to the native TCR (9–11). The mechanism underlying this profound defect in antigen sensitivity, which is seen with both proximal (10, 11) and distal readouts of T cell activation (9), remains unclear.

One approach to improving CAR function has focused on varying the stalk/hinge region and/or the cytoplasmic signaling domains. There are several commonly used hinges, including from CD8a, CD28, and IgG1. Most CARs use the cytoplasmic domain of the TCR *ζ*-chain for signaling, either alone (1st generation) or in combination with the CD28 signalling or 4-1BB cytoplasmic signaling domains (2nd generation) (12–15). A study comparing the ability of several of these CARs to kill target cells with very low antigen densities found that the CARs that performed best had the CD28 hinge and the signaling domain from *ζ*-chain, either alone or in combination with the CD28 domain (16). Other studies have replaced the TCR *ζ*-chain with the cytoplasmic chain of the CD3*ε* subunit of the TCR/CD3 complex (11, 17, 18).

A second approach to improving CAR function has focused on exploiting all the signaling domains present in the TCR/CD3 complex. For example, eTruC receptors fuse the scFv directly with the extracellular domain of CD3*ε* (19), whereas STARs (also called HIT receptors) replace the variable domains of the TCR with the scFv variable domains (20, 21). Using a xenograft carcinoma model with EGFR as the target antigen, a STAR outperformed an eTruC, and both outperformed CARs (20). The precise mechanisms underlying these performance differences are unclear.

The TCR is known to have remarkable antigen sensitivity; it is able to recognize even a single-peptide major histocompatibility complex (pMHC) on cells (22). Diverse mechanisms have been shown to contribute to this sensitivity (23). These include having multiple immunoreceptor tyrosine-based activation (ITAMs) (24, 25), using the TCR coreceptors CD4 or CD8 (26, 27), and exploiting TCR accessory receptors such as LFA-1 (28) and CD2 (29). Despite the known importance of accessory receptors in enhancing TCR antigen sensitivity, their contribution to CAR antigen sensitivity has not been measured. Interestingly, CD2 has been shown to affect T cell activation by 1st generation CARs, but its impact on CAR antigen sensitivity is presently unknown (30).

Here, we take advantage of a shared pMHC antigen ligand to directly compare the antigen sensitivity of CARs with the native TCR. We show that, while CARs exhibit a > 100-fold lower antigen sensitivity than TCRs to antigen presented on cells, they exhibit nearly identical sensitivities to antigen in the absence of accessory receptor ligands. We then demonstrate that engagement of accessory receptors only modestly increases the sensitivity of CARs to antigen, despite dramatically enhancing the sensitivity of the TCR. Finally, we show that TruCs and STARs/HITs have greater antigen sensitivity than CARs and that this correlates with their ability to exploit CD2 to enhance this sensitivity. Our work helps explain the profound defect in CAR sensitivity and suggests ways to improve it for therapeutic purposes.

## Results

### Standard CAR Designs Exhibit Reduced Sensitivity Compared to the TCR When Antigen is Presented on APCs but Not When Presented in Isolation.

**Fig. 1.**
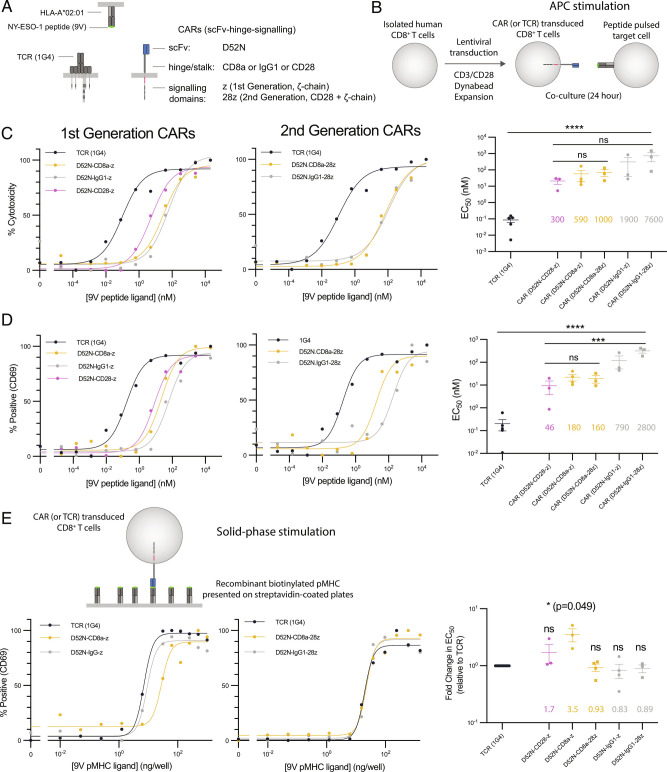
CARs show reduced sensitivity compared to the TCR when antigen is presented on APCs but not when presented as purified protein. (*A*) Schematic of antigen receptors. The 1G4 TCR and the D52N scFv both recognize the 9V NY-ESO-1 peptide antigen presented on HLA-A*02:01. CARs using the CD8a hinge contain the CD8a transmembrane domain, whereas CARs using the IgG1 or CD28 hinges contain the CD28 transmembrane domain. (*B*) Schematic of APC stimulation system. (*C* and *D*) Representative dose–response showing (*C*) cytotoxicity by LDH release and (*D*) surface expression of CD69 for the TCR and the indicated CARs along with EC_50_ values from at least three independent experiments determined by fitting a Hill function to each dose–response curve. (*E*) Representative dose–response when the purified biotinylated 9V pMHC ligand is presented on streptavidin-coated plates (*Left* two plots) and EC_50_ values from at least three independent experiments (*Right*). The EC_50_ values are compared using (*C* and *D*) one-way ANOVA or (*E*) one-sample *t*-test for a hypothetical mean of 1.0 on log-transformed values. *P-value ≤ 0.05, **P-value ≤ 0.01, ***P-value ≤ 0.001, ****P-value ≤ 0.0001.

To compare the antigen sensitivity of TCRs and CARs, we utilized the C9V variant (9V) of the cancer testis peptide antigen expressed on HLA-A*02:01 because it is recognized by both the 1G4 TCR (31, 32) and the D52N scFv (33) (Fig. 1*A*). While D52N binds to pMHC in a similar orientation to the 1G4 TCR (34), it binds 9V pMHC with a higher affinity (33). We produced five CAR designs by fusing the D52N scFv to either the CD28, CD8a, or IgG1 hinge coupled to either the TCR *ζ*-chain alone (1st generation) or in combination with the CD28 signaling chain (2nd generation) (*SI Appendix*, Fig. S1).

Using a standard protocol similar to those employed in ACT (35), we transduced primary human CD8^+^ T cells with each antigen receptor and expanded them in vitro before coculturing them with the HLA-A*02:01+ T2 target cell line pulsed with different concentrations of antigen (Fig. 1*B*). We found that T cells expressing the 1G4 TCR were able to kill target cells (as measured by LDH release) at 300- to 7600-fold lower concentration of peptide antigen compared to CARs (Fig. 1*C*). We observed similar results when measuring the upregulation of the CD69 activation marker, albeit with lower 46- to changes (Fig. 1*D*). The large antigen sensitivity differences between the TCR and CARs could not readily be explained by receptor surface expression because the CARs were expressed at the same (or higher) levels than the TCR, as measured by pMHC tetramer binding (*SI Appendix*, Fig. S2). This > 100-fold higher sensitivity of the TCR is consistent with two previous reports (9, 10) that utilized different hinges and different signaling chains (2nd generation CARs with 4-1BB coupled to the *ζ*-chain). Our finding that a CAR with the CD28 hinge had the highest antigen sensitivity is also consistent with a previous report (16). Taken together, these results validate our antigen receptor system and suggest that reduced antigen sensitivity is a general feature of CARs.

We next compared antigen sensitivity of TCR and CARs when presented with plate immobilised pMHC (Fig. 1*E*). This reductionist system allows precise control of TCR and accessory receptor ligands (32, 36–39). In striking contrast to the difference in sensitivity when antigen was presented on cells, the TCR and CARs displayed similar antigen sensitivities when recognizing purified antigen, with the largest difference being 3.5-fold (Fig. 1*E*).

### Ligands to the Adhesion Receptors CD2 and LFA-1 Increase the Antigen Sensitivity Difference Between the TCR and CARs.

**Fig. 2.**
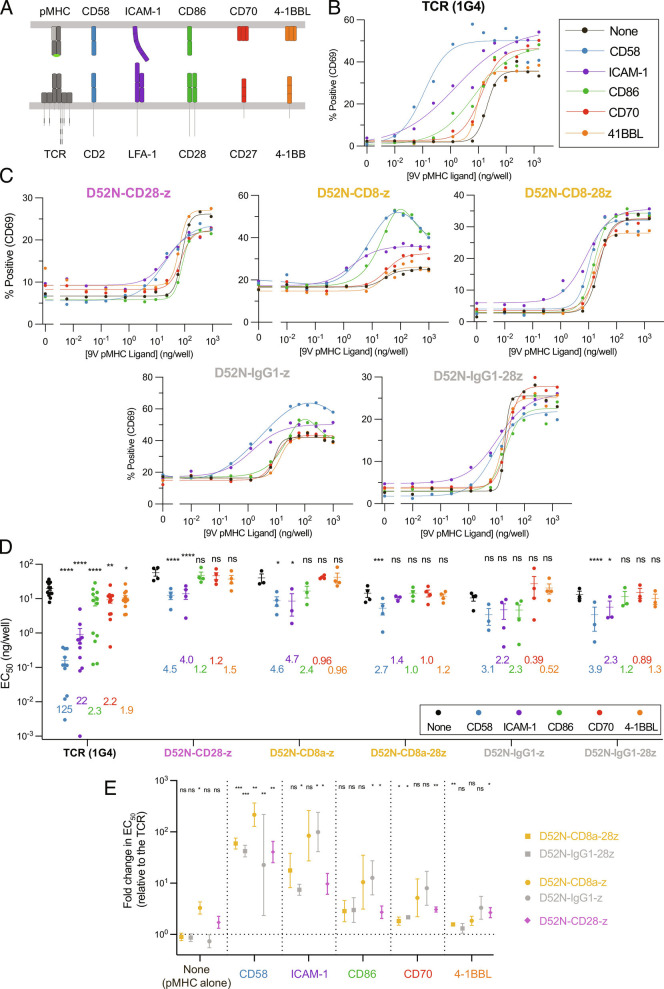
Systematic engagement of accessory receptors identifies that CARs are inefficient at exploiting the adhesion receptors CD2 and LFA-1 relative to the TCR. (*A*) Schematic of accessory receptors and their ligands. (*B* and *C*) Representative dose–response curves showing T cell activation by upregulation of surface CD69 measured by flow cytometry after 24 h using the solid-phase stimulation assay. T cells were presented with purified pMHC alone (“None”) or with a fixed concentration of 250 ng/well of the indicated accessory receptor ligand (colors) for the (*B*) TCR and (*C*) the indicated CARs. (*D*) The EC_50_values for the indicated antigen receptor and purified ligand condition were obtained by fitting a Hill function to each dose–response curve. Individual EC_50_values for each antigen receptor are from an independent experiment (N ≥ 3). The numbers indicate the fold-change in EC_50_induced by the accessory receptor ligand relative to pMHC alone (“None”), and statistical significance is determined by a paired *t*-test on log-transformed data. (*E*) The data in (*D*) are presented in a different format showing the fold-change in EC_50_between the TCR and the indicated CAR for pMHC alone or the indicated accessory receptor ligand. The fold-change is compared using a one-sample *t*-test to a hypothetical value of 0 on log-transformed data. *P-value ≤ 0.05, **P-value ≤ 0.01, ***P-value ≤ 0.001, ****P-value ≤ 0.0001.

Our finding that the ≳100-fold higher sensitivity of TCR compared to CARs is eliminated in a reductionist system provided an opportunity to explore the underlying mechanism. A key difference between cells and our reduced system is the presence of accessory receptor/ligand interactions involving T cell accessory receptors CD2, LFA-1, CD28, CD27, and (Fig. 2*A*). To investigate whether engagement of these receptors can account for the sensitivity differences, we tested their ability to increase antigen sensitivity by including, alongside pMHC, purified forms of their ligands at a concentration of (250 ng/well) previously shown to enhance T cell responses (32, 38, 40).

While ligands for CD2 (CD58), LFA-1 (ICAM-1), CD28 (CD86), CD27 (CD70), and 4-1BB (4-1BBL) all enhanced TCR antigen sensitivity, only CD58 and ICAM-1 increased CAR sensitivity (Fig. 2*B*–*D*). CD58 and ICAM-1 produced the largest increases in TCR antigen sensitivity (125- and, respectively), while CD86, CD70, and 4-1BBL produced much smaller increases (Fig. 2*D*). Strikingly, CARs were much less efficient at exploiting these ligands than the TCR, with only CD58 and ICAM-1 increasing sensitivity and only by 1.4 to 4.7 fold (Fig. 2*D*). When performing independent experiments (Fig. 2*D*, individual EC_50_values), we isolated and transduced T cells from each donor with the TCR and one or more CARs. By always including the TCR, we could express the antigen sensitivity of all CARs relative to the TCR (Fig. 2*E*). This confirmed that the TCR and CARs were similarly sensitive when stimulated with only pMHC, while the TCR was more sensitive when ligands to accessory receptors were present, with the largest differences observed when including ligands to CD2 or LFA-1.

To confirm these findings with another readout of T cell activation, we measured production of the inflammatory cytokine IFN*γ*. As observed when using CD69 upregulation as a readout, accessory receptor ligands increased TCR sensitivity much more than CAR sensitivity, with CD2 ligands producing the biggest increases (*SI Appendix*, Fig. S3).

It has previously been reported that tonic signaling by CARs can lead to T cell dysfunction/exhaustion by various mechanisms, including altering the expression of surface receptors (41–43), raising the possibility that tonic CAR signaling abrogates antigen sensitivity. To investigate this, we first measure expression levels of accessory receptors (*SI Appendix*, Fig. S4*A*) and exhaustion markers LAG-3, PD-1, and TIM-3 (*SI Appendix*, Fig. S4*B*). These were indistinguishable between TCR and CAR-transduced T cells, except for a < 2-fold increase in TIM-3. Next, we showed that transduction of a CAR did not affect the sensitivity of an orthogonal TCR recognizing a viral peptide, with or without the CD2 ligand (*SI Appendix*, Fig. S4*C*). This ruled out tonic signaling as an explanation for the defect in CAR antigen sensitivity.

Supraphysiological affinities can impair TCR signaling and reduce antigen sensitivity (37, 44, 45), and lowering the affinity of CARs has been shown to improve their in vivo activity (46). It follows that the higher affinity of the D52N scFv than the 1G4 TCR for the 9V (∼50-fold higher at 37 °C, *SI Appendix*, Fig. S5*A*) could account for the defect in CAR sensitivity. To investigate this, we identified a lower-affinity pMHC that bound the D52N scFv with the same affinity that the 1G4 TCR binds the 9V pMHC (*SI Appendix*, Fig. S5*A*; 4A pMHC). When using these matched affinity pMHC antigens, the difference in antigen sensitivity between the TCR- and CAR-transduced T cells was increased rather than decreased (*SI Appendix*, Fig. S5 *B*–*E*), demonstrating that the higher affinity of the CAR for antigen cannot account for its lower sensitivity.

The CD8 coreceptor binds pMHC, raising the possibility that it contributes to antigen sensitivity differences between the TCR and CARs. To investigate this, we repeated the solid-phase stimulation assay using a pMHC variant with point mutations that abolish CD8 binding (47) (*SI Appendix*, Fig. S6). Eliminating CD8 binding had no impact on the CAR sensitivity and only a modest impact for the TCR when recognizing the 9V pMHC. Interestingly, eliminating CD8 binding abolished TCR recognition of the very low-affinity 4A pMHC, consistent with previous work showing that CD8 has disproportionate impact on recognition of low-affinity antigens by TCR (48, 49). These findings show that CD8 binding cannot account for the profound difference in TCR vs CAR antigen sensitivity.

In summary, the antigen sensitivities of the TCR and CARs are similar when presented with purified antigen in isolation, and antigen sensitivity of the TCR is enhanced far more than CARs when including ligands for accessory receptors, especially CD2. This difference in TCR and CAR antigen sensitivity is not a result of differences in tonic signaling, affinity for antigen, or the contribution of the CD8 coreceptor.

### Abrogating the CD2 and LFA-1 Interaction Reduces the Antigen Sensitivity Difference Between the TCR and CARs.

**Fig. 3.**
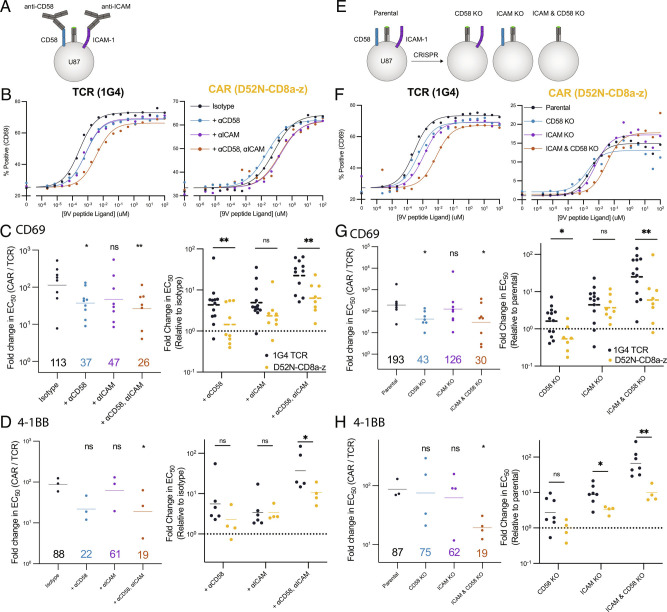
Abrogating the CD2 and LFA-1 adhesion interaction disproportionately impacts the antigen sensitivity of the TCR compared to the CAR. (*A*) Schematic of CD58 and ICAM-1 blocking experiment on the HLA-A2+ glioblastoma U87 target cell line. (*B*) Representative dose–response curves for the indicated blocking conditions for the TCR (*Left*) and CAR (*Right*). (*C* and *D*) Fold-change in EC_50_between the CAR and TCR (*Left*) or relative to the isotype (*Right*) for (*C*) CD69 and (*D*) 4-1BB upregulation. (*E*) Schematic of CD58 and ICAM-1 knockout experiments. (*F*) Representative dose–response curves for the indicated target cell lines for the TCR (*Left*) and CAR (*Right*). (*G* and *H*) Fold-change in EC_50_between the CAR and TCR ((*Left*) or relative to the isotype (*Right*) for (*G*) CD69 and (*H*) 4-1BB. Individual EC_50_values for CD69 or 4-1BB are determined by a fit to the dose–response curve from at least three independent experiments (each data point in *C*, *D*, *G*, and*H* is from an independent experiment). The fold-change between the TCR and CAR is compared using a two-sample *t*-test to the isotype or parental line condition (*Left* in *C*, *D*, *G*, and *H*) or directly between the TCR and CAR (*Right* in *C*, *D*, *G*, and *H*) on log-transformed values. *P-value ≤ 0.05, **P-value ≤ 0.01, ***P-value ≤ 0.001, ****P-value ≤ 0.0001.

Our results using an artificial system indicate that the antigen sensitivities of the TCR and CARs were similar when recognizing purified antigen in isolation but exhibited large differences with the addition of purified ligands to CD2 or LFA-1 (Figs. 1 and 2). To investigate the role of these accessory adhesion receptor interactions in target cell recognition, we utilized the HLA-A*02:01+ U87 glioblastoma cell line, which expresses CD58 and ICAM-1 (*SI Appendix*, Fig. S7 *A* and *B*). We compared the TCR to the first-generation CD8a hinge CAR (D52N-CD8a-z) because this CAR displayed the largest increase in antigen sensitivity when adding purified CD58 and ICAM-1 (Fig. 2*D*). We used blocking antibodies (Fig. 3*A*–*D*) or CRISPR (*SI Appendix*, Fig. S7 *A* and *B* and Fig. 3*E*–*H*) to abrogate CD58 and/or ICAM-1 engagement and quantitated the effect on T cell sensitivity to pMHC antigen by measuring CD69 and 4-1BB expression. There was a profound ∼100-fold difference in antigen sensitivity between TCR and CAR-transduced T cells, as shown above with T2 cell targets, which decreased to ∼20-fold when abrogating both the CD2 and LFA-1 interaction (Fig. 3*C*, *D*, *G*, and *H*, *Left*). This decrease was mainly the result of a decrease in antigen sensitivity of the TCR (Fig. 3*C*, *D*, *G*, and *H*, *Right*). The fact that the antigen sensitivity of the TCR remained 20-fold higher than the CAR indicates that other mechanisms, including perhaps other ligand interactions, contribute to its higher sensitivity. In support of this, the U87 cell line expresses LFA-1 ligands other than ICAM-1 (*SI Appendix*, Fig. S7*C*). Lastly, we reproduced these findings using the Nalm6 leukemia cell line (*SI Appendix*, Fig. S8). In summary, our experiments with antigen presented on artificial surfaces or cells suggest that TCRs have higher antigen sensitivities than CARs because they exploit the accessory receptors such as CD2 and LFA-1 more efficiently.

### STARs Display TCR-like Antigen Sensitivity Outperforming TRuCs and CARs by Efficiently Exploiting Adhesion Receptors.

**Fig. 4.**
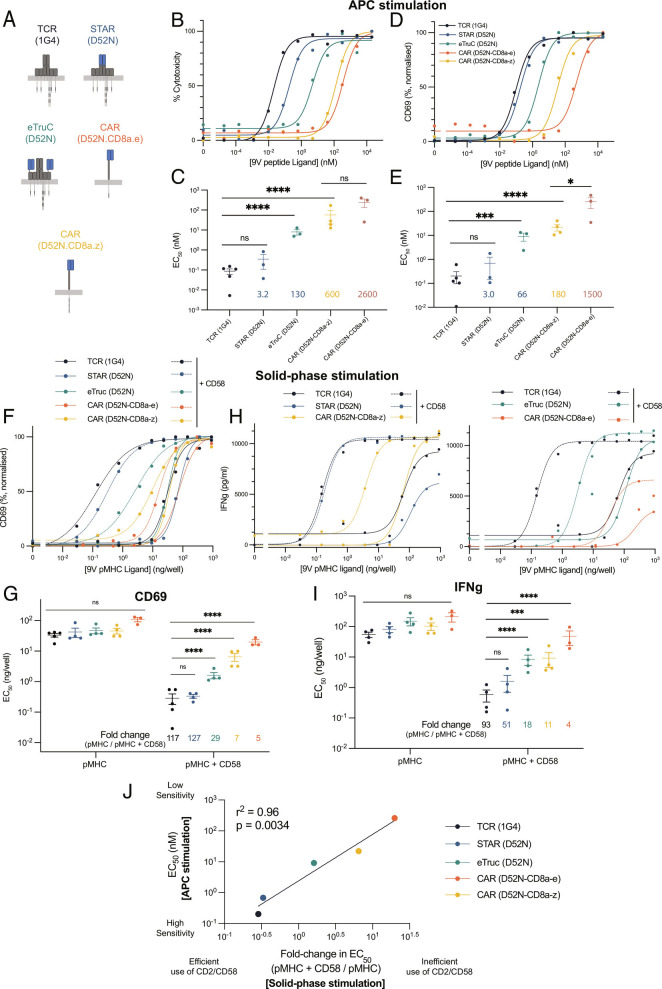
The ability of TCR-like chimeric antigen receptors to recapitulate the sensitivity of the TCR depends on the efficiency with which they are able to exploit the CD2 adhesion interaction. (*A*) Schematic of “TCR-like” engineered antigen receptors. (*B*–*E*) T cells expressing the indicated antigen receptor were cocultured with T2 target cells pulsed with different peptide antigen concentrations for 8 h. Representative dose–response (*Top*) and fitted EC_50_values from at least three independent experiments (*Bottom*) are shown for (*B* and *C*) cytotoxicity (measured by LDH release) and (*D* and *E*) CD69 upregulation. (*F*–*I*) T cells expressing the indicated antigen receptor were stimulated by a titration of purified pMHC alone (solid lines) or in combination with a fixed concentration of purified CD58 (dashed lines). Representative (*F* and *H*) dose–response curves and (*G* and *I*) fitted EC_50_values from at least three independent experiments for (*F* and *G*) CD69 upregulation and (*H* and *I*) IFN*γ* production. (*J*) The averaged EC_50_values for CD69 upregulation from the APC stimulation assay (from panel *C*) are plotted over the averaged fold-change in EC_50_for CD69 induced by the addition of CD58 from the solid-phase stimulation assay (from panel *G*). The EC_50_values are compared using a one-way ANOVA on log-transformed values (*C*, *E*, *G*, and *I*). *P-value ≤ 0.05, **P-value ≤ 0.01, ***P-value ≤ 0.001, ****P-value ≤ 0.0001.

The ability of the TCR to exploit adhesion interactions has been shown to depend on both TCR signaling (50) and structural features of the TCR/pMHC interaction (23). The fact that conventional CARs lack signaling motifs present in the native TCR/CD3 complex has motivated the construction of additional chimeric receptors. These include CARs containing cytoplasmic signaling chain of CD3*ε* (17, 18); TruCs, in which the scFv is fused to the extracellular domain of CD3*ε* and assembled into a complete TCR complex (19); and STARs, in which the TCR *α* and *β* chain variable domains are replaced with the antibody variable domains (20). These chimeric receptors increasingly resemble the native TCR complex in terms of signaling components and structure (Fig. 4*A*).

To directly compare the antigen sensitivities of these receptors using our system, we generated versions containing the D52N variable domains (Fig. 4*A*). The CAR and eTruC incorporated the D52N scFv, which contains a linker between the variable domains (*SI Appendix*, Fig. S5). The STAR incorporates the D52N variable domains into separate chains (*SI Appendix*, Fig. S1). Because this lacks the linker present in the scFv, we generated purified STAR and confirmed that it bound the pMHC, albeit with a 10-fold lower affinity than scFv (*SI Appendix*, Fig. S9). When transduced into T cells, the surface expression of these chimeric receptors was indistinguishable from the 1G4 TCR (*SI Appendix*, Fig. S2*D*, last three columns).

We next measured the sensitivity of these chimeric receptors to antigen presented on cells (APC stimulation) using target cell killing and CD69 upregulation as readouts (Fig. 4). We found that the STAR performed identically to the TCR, while the eTruC was intermediate between them and the standard *ζ*-chain CAR. The *ε*-chain CAR was less sensitive than the *ζ*-chain CAR. To determine whether adhesion interactions can account for these differences, we examined the impact of CD2 engagement on sensitivity to antigen presented on plates (solid-phase stimulation), using CD69 upregulation and cytokine production as readouts (Fig. 4*F*–*I*). As before, we found nearly identical antigen sensitivities for all antigen receptors when presented with purified antigen alone. Addition of the CD2 ligand, CD58 increased antigen sensitivity by different amounts, mirroring the antigen hierarchy observed with APC stimulation. Indeed, the efficiency with which an antigen receptor was able to exploit CD2 engagement directly predicted its antigen sensitivity measured using cells (Fig. 4*J*). We repeated the solid-phase stimulation assay using the LFA-1 ligand ICAM-1 and found a similar conclusion, albeit with lower fold-changes (*SI Appendix*, Fig. S10). We next compared the antigen sensitivity using the panel of U87 target cells finding that the difference in antigen sensitivity between the TCR and both the eTruC and *ε*-chain CAR is reduced when abrogating the CD2 and LFA-1 adhesion interactions (*SI Appendix*, Fig. S11). Lastly, these conclusions were reproduced when using CD4+ T cells (*SI Appendix*, Fig. S12). Taken together, these results suggest that the antigen sensitivity of these TCR-like chimeric antigen receptors depends on their ability to exploit the CD2/CD58 and LFA-1/ICAM-1 adhesion interactions.

### CARs Fail to Exploit the Ability of Adhesion Receptors to Enhance CAR Downregulation by Antigen Engagement.

We next investigated the ability of CD2 and LFA-1 to enhance antigen engagement by CARs. TCR/pMHC engagement has been quantified by measuring downregulation of the TCR from the T cell surface (51), and it has been shown using this approach that TCR/pMHC engagement is enhanced by CD2 or LFA-1, when using either APCs (28, 29) or purified proteins (32, 39).

**Fig. 5.**
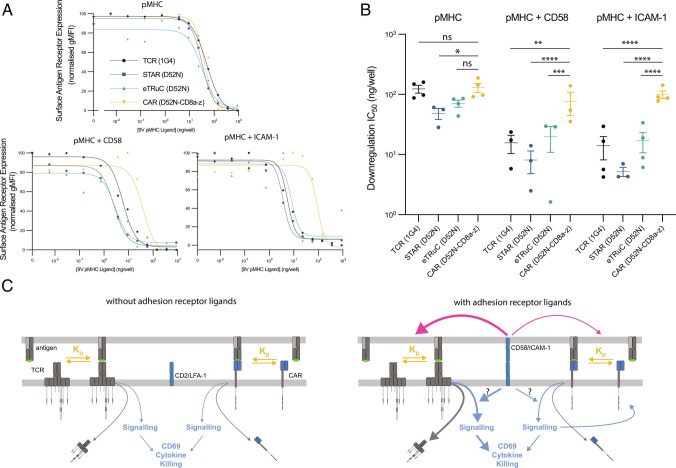
Adhesion receptors more efficiently enhance antigen engagement for the TCR compared with the eTruC and CAR. (*A* and *B*) Effect of pMHC with or without CD58 or ICAM-1 on surface antigen receptor expression, as determined by pMHC tetramers. (*A*) Representative curves and (*B*) fitted IC_50_ values from at least three independent experiments. All data are normalized to surface expression without pMHC. All comparisons are made using a one-way ANOVA on log-transformed values. *P-value ≤ 0.05, **P-value ≤ 0.01, ***P-value ≤ 0.001, ****P-value ≤ 0.0001. (*C*) Model showing similar performance of a TCR and a CAR in the absence of ligands for adhesion receptors (*Left*) and superior enhancement of TCR vs. CAR antigen engagement (red arrows) and signaling (blue arrows) by adhesion receptor ligands (*Right*).

In the absence of CD2 and LFA-1 ligands, we find that the TCR, STAR, eTruC, and CAR are down-regulated at similar concentrations of pMHC (Fig. 5 *A* and *B*). However, the addition of CD58 or ICAM-1 resulted in increased downregulation of the TCR, STAR, and eTruC compared to the CAR (Fig. 5 *A* and *B*). We performed these experiments by adding Brefeldin A because, as observed by others (52), there was a large increase in CAR surface expression in this assay (*SI Appendix*, Fig. S13). The pMHC concentrations that stimulate CAR upregulation (∼1 to 10 ng/well) are similar to the concentrations required to up-regulate CD69 and IFN in the presence of CD58 or ICAM-1 (Fig. 4*F*–*I*), suggesting that this upregulation is regulated, at least in part, by CAR signaling. Lastly, both basal (*SI Appendix*, Fig. S4*A*) and antigen-stimulated (*SI Appendix*, Fig. S14) expressions of CD2 and LFA-1 were similar between T cells expressing different antigen receptors.

Taken together, these results suggest that the inability of CD2 and LFA-1 to enhance the sensitivity of CARs to antigen is consistent with a mechanism whereby they fail to enhance the engagement of antigen by CARs.

## Discussion

The ability of CAR T cells to control cancer cell mutants that express low antigen levels will depend on their sensitivity to antigen (1, 2, 6). We have shown here that several CAR formats, including 1st and 2nd generation CARs, have a > 100-fold lower sensitivity to antigen than the TCR. We further showed that this low sensitivity is the result of a failure of these CARs to efficiently exploit the adhesion receptors CD2 and, to a lesser extent,. Finally, we show that this failure is reversed when chimeric antigen receptors are redesigned to match more closely the native TCR structure.

In principle, accessory receptors can enhance antigen receptor sensitivity by enhancing their engagement of antigen and/or by enhancing their signaling (Fig. 5*C*). Our observation that CD2 and LFA-1 are less effective at enhancing CAR down-modulation than TCR down-modulation (Fig. 5 *A* and *B*) suggests a defect in enhancing engagement of antigen. Although these results are consistent with a defect in the ability of CD2 and LFA-1 to enhance CAR antigen engagement, we have not excluded an additional defect in their ability to modulate CAR signaling (Fig. 5*C*). Indeed, previous studies have shown that multiple ITAMs are important for TCR antigen sensitivity (25, 53, 54). However, a recent study suggests that simply increasing the number of ITAMs in a CAR does not improve antigen sensitivity (55). Nonetheless, our findings here are consistent with the reduced number/variety of ITAMs in CARs contributing to their defect in sensitivity to antigen.

Because it is difficult to vary the CAR target antigen concentrations on cells, only a handful of studies have directly measured the antigen sensitivity of CARs. Consistent with our work, these studies reported a ∼100 to 1000-fold defect in CAR antigen sensitivity compared to the TCR. Using a CAR containing the variable domains of a TCR, Harris et al. (9) showed that both 1st and 2nd generation CARs exhibited a ∼100-fold lower antigen sensitivity than the native TCR. Wang et al. (56) found similar defects when using primary T cells and, consistent with our findings, observed only modest impacts of CD28 engagement on the antigen sensitivity of TCR and CARs (Fig. 2). Gudipati et al. (10) report similar findings using antigens presented on planar bilayers, which contained ICAM-1, consistent with our result in our solid-phase stimulation system when ICAM-1 is included (Fig. 2*E*). When comparing the antigen sensitivities of different CARs, Majzner et al. (16) found that CARs with the CD28 hinge produced the highest sensitivity. This was achieved with a first generation CAR and thus did not require signaling by CD28 or 4-1BB. We also found that the CD28 hinge CAR produced the highest antigen sensitivity among the CARs we tested (Fig. 1) and that CD28 or 4-1BB ligands produced only modest enhancements in antigen sensitivity (Fig. 2). Lastly, Salter et al. (11) showed that incorporation of a proline-rich region or the GRB2 SH2 domain into a second-generation CAR with signaling domain can enhance antigen sensitivity but these CARs continued to display lower antigen sensitivity than the second-generation CARs with CD28 signaling domains. We note that, while costimulation signals have a modest impact on antigen sensitivity, they are nevertheless critical for in vivo tumor control presumably because they improve CAR-T cell persistence and increase cytokine production (57). Thus, our results are consistent with the previous studies and extend them by identifying inefficient exploitation of adhesion receptors as a cause of the reduced antigen sensitivity of CARs.

While studies in mice suggested a modest role for CD2 (29, 58), it is clearly important in human T cell function (32, 59), including elimination of cancerous (60, 61) and virus-infected (62) cells. Defects in CD58 (either loss of expression or mutations) have been reported in B cell and T cell lymphomas (63–65), and CD2 expression on tumor-infiltrating T cells has been shown to correlate with their function in several cancers (66). Patients with B cell lymphomas with CD58 defects showed reduced progression-free survival when treated with axicabtagene ciloleucel CAR-T cell therapy (67). This implies that even the reduced ability of CARs to exploit CD2 can still impact in vivo efficacy. Our finding that TruCs and STARs can more efficiently exploit CD2 to achieve higher antigen sensitivities is consistent with the finding that a STAR outperformed an eTruC and that both outperformed CARs in an in vivo xenograft tumor model (20). The recent finding that poor LFA-1 adhesion contributes to impaired killing of solid tumors by CAR T cells (68) is consistent with our results showing that CARs are much less efficient at exploiting LFA-1 compared to the TCR. Taken together, this highlights the critical importance of adhesion receptor interactions in CAR T cell function.

Although high antigen sensitivity is often beneficial, there are scenarios where low antigen sensitivity is desirable, such as when the target antigen is expressed at high levels on cancer cells and low levels on normal cells (69, 70). It has previously been shown that antigen sensitivity can be tuned by changing the affinity of the CAR (46, 70, 71) or by using transcriptional circuits (72). The results presented here show that antigen sensitivity can also be tuned by altering the CAR architecture. For example, the sensitivity hierarchy that we observe [STARS > TruCs > CAR (CD28 hinge) > CAR (CD8a hinge) > CAR (IgG1 hinge)] suggests that standard CARs may be preferred for targeting cancers that overexpress antigens also expressed on normal cells. In contrast, STARs may be preferred in cancers with low levels of target antigen or which commonly escape by reducing expression of the antigen. Importantly, STARs would remain susceptible to immune evasion by cancer cells losing expression of CD58 and/or ICAM-1. An advantage of tuning antigen sensitivity by changing the CAR architecture is that changes to the recognition domain are not required, reducing the risk of inadvertently altering its specificity.

The TCR, and indeed CARs, belong to a large and diverse group of surface receptors known as immunoreceptors or noncatalytic tyrosine-phosphorylated receptors (NTRs) (73). The mechanism by which these receptors convert extracellular ligand binding into intracellular signaling, known as receptor triggering, remains debated. In the case of the TCR, allosteric conformational changes have been proposed as a triggering mechanism (74). While previous work has shown that grafting antibody variable domains to replace TCR variable domains produces a functional receptor (20, 75), it was unclear how this receptor compared to the native TCR. Our results here show that this chimeric receptor (STAR/HIT) is indistinguishable from the TCR in terms of antigen sensitivity. This observation is difficult to reconcile with allosteric models of TCR activation given the very limited conservation between antibody and TCR variable domains. Our results are, however, compatible with conformational changes induced by mechanical pulling forces, as well as models that do not require conformational change, such as the kinetic segregation model, which postulates that ligand binding segregates NTRs from inhibitory tyrosine phosphatases (73). Indeed, a recent study strongly suggests that CARs trigger using the kinetic segregation mechanism (76).

In conclusion, we show that it is possible to engineer chimeric receptors with the same antigen sensitivities as the TCR and that this requires that they efficiently exploit the adhesion receptors CD2 and. This suggests a simple way to tune antigen sensitivity in order to optimize the functional effect of T cells. While our results suggest a strategy to reduce immune escape, it does not eliminate it because cancers can abolish expression of the target antigen. Other strategies, including targeting multiple antigens, may be necessary to further reduce escape (77). There is increasing interest in redirecting other immune cells, such as macrophages, using chimeric antigen receptors (78, 79). Since these cell do not usually express CD2, our work suggests that introducing CD2 or another adhesion receptor may be necessary to achieve the same remarkable antigen sensitivity as the TCR.

## Materials and Methods

The peptide ligands were commercially synthesized and the protein ligands were purified from *E. coli* or HEK293 cells to produce pMHC or accessory receptor ligands (CD58, ICAM-1, CD86, CD70, and 4-1BBL), respectively. T cell production followed a standard lentiviral transduction and proliferation protocol. T cell activation was assessed using either T2 or U87 for the APC stimulation or by coupling purified biotinylated ligands to commercial streptavidin plates. To assess antigen affinity and kinetics, a BIAcore T200 instrument operating at 37° was used. The detailed *Material and Methods* section, including statistical information on independent *Experiments and Data Analysis*, can be found in the *SI Appendix*.

## Supplementary Material

Appendix 01 (PDF)

## Data Availability

All study data are included in the article and/or *SI Appendix*.
