# Supplementary material for: Inefficient exploitation of accessory receptors reduces the sensitivity of chimeric antigen receptors
Source: Proc Natl Acad Sci U S A. 2023 Jan 4;120(2):e2216352120. doi: 10.1073/pnas.2216352120 (PMC9926289; doi:10.1073/pnas.2216352120)
Supplement: Supplementary file 1 — Appendix 01 (PDF) [file pnas.2216352120.sapp.pdf]

PNAS
www.pnas.org

Supplementary Information for

Inefficient exploitation of accessory receptors reduces the sensitivity of chimeric antigen receptors

Jake Burton[1], Jesús A. Siller-Farfán[1], Johannes Pettmann[1], Benjamin Salzer[1], Mikhail Kutuzov[1], P. Anton van der Merwe[1], and Omer Dushek[1]

[1] Sir William Dunn School of Pathology, University of Oxford, OX1 3RE, Oxford, UK

Corresponding Author: Omer Dushek
E-mail: omer.dushek@path.ox.ac.uk

This PDF file includes:

Supplementary text
Figs. S1 to S14
SI References

Supporting Information Text

Materials & Methods

Peptides. Peptides were synthesised at a purity of >95% (Peptide Protein Research, UK). 9V refers to a peptide derived from NY-ESO$_{157–165}$ (`SLLMWITQV`), 4A is derived from the same sequence (`SLLAWITQV`), and SL9 refers to a peptide from HIV p17 GAG$_{77-85}$ (`SLYNTVATL`).

Protein production. HLA-A*02:01 heavy chain (UniProt residues 25–298) with a C-terminal BirA tag and $_2$-microglobulin were expressed as inclusion bodies in *E.coli*, refolded *in vitro* as described in (1) together with the relevant peptide variants, and purified using size-exclusion chromatography on a Superdex S75 column (GE Healthcare, USA) in HBS-EP buffer (10 mM M HEPES pH 7.4, 150 mM NaCl, 3 mM EDTA, 0.005% v/v Tween-20). Purified pMHC was biotinylated using the BirA enzyme (Avidity, USA).

His-tagged, soluble extracellular domain (ECD) of human CD58 was produced either in Freestyle 293F suspension cells (Thermo Fisher) or adherent HEK 293T cells. His-tagged, soluble versions of the ECD of human ICAM1, 41BBL, CD70 and CD86 were produced using adherent HEK 293T cells. Freestyle 293F suspension cells were transfected using Freestyle MAX reagent, as previously reported (2). Adherent HEK 293T cells were transfected using Roche X-tremeGENE HP transfection reagent following the manufacturer's protocol. In both cases the resulting supernatant was filtered with a 0.45 μm filter and proteins were then purified using Ni-NTA agarose columns. Biotinylation was either performed *in vitro* after purification, or *in situ* by co-transfection (final proportion 10%) of a secreted BirA and adding 100 μM D-biotin to the growth media. Further purification and excess biotin removal was performed by size exclusion chromatography in HBS-EP.

D52N chains were produced as inclusion bodies in E. coli and refolded in vitro as described in (3), except that inclusion bodies were solubilised in 20 mM Tris-HCl (pH 8.0), 8 M urea, 2 mM DTT, refolding buffer contained 150 mM Tris-HCl (pH 8.0), 3 M urea, 200 mM Arg-HCl, 0.5 mM EDTA, 0.1 mM PMSF, and the refolding mixture was dialysed against 10 mM Tris-HCl (pH 8.5). The D52N dimer was purified on anion-exchange chromatography on a HiTrap Q column, followed by size-exclusion chromatography on a Superdex S200 column (both from GE Healthcare).

All purified proteins were aliquoted and stored at -80 until use.

Lentiviral production. HEK 293T cells were seeded in DMEM supplemented with 10% FBS and 1% penicilin/streptomycin in 6-well plates to reach 60–80% confluency on the following day. Cells were transfected with 0.25 pRSV-Rev (Addgene, #12253), 0.53 μg pMDLg/pRRE (Addgene, #12251), 0.35 μg pMD2.G (Addgene, #12259), and 0.8 μg of transfer plasmid using 5.8 X-tremeGENE HP (Roche). Media was replaced after 16 hours and supernatant harvested after a further 24 hours by filtering through a 0.45 cellulose acetate filter. Supernatant from one well of a 6-well plate was used to transduce 1 million T cells.

T cell production. Human CD8$^+$ T cells were isolated from leukocyte cones purchased from the National Health Service's (UK) Blood and Transplantation service. Isolation was performed using negative selection. Briefly, blood samples were incubated with Rosette-Sep Human CD8$^+$ enrichment cocktail (Stemcell) at 150 for 20 minutes. This was followed by a 3.1 fold dilution with PBS before layering on Ficoll Paque Plus (GE) at a 0.8:1.0 ficoll to sample ratio. Ficoll-Sample preparation was spun at 1200for 20 minutes at room temperature. Buffy coats were collected, washed and isolated cells counted. Cells were resuspended in complete RMPI (RPMI supplemented with 10% v/v FBS, 100 penicillin, 100 streptomycin) with 50U of IL-2 (PeproTech) and CD3/CD28 Human T-activator Dynabeads (Thermo Fisher) at a 1:1 bead to cell ratio. At all times isolated human CD8$^+$ T cells were cultured at 37 and 5% CO_2.

1 in 1 of media were subsequently transduced on the following day using lentivirus encoding for the various constructs (e.g., TCR), per the section on lentiviral transduction. On days 2 and 4 post-transduction, 1 of media was exchanged and IL-2 was added to a final concentration of 50U. Dynabeads were magnetically removed on day 5 post-transduction. T cells were further cultured at a density of 1 and supplemented with 50U IL-2 every other day. T cells were used between 10 and 16 days after transduction.

APC stimulation (co-culture with T2 cells). T2 cells were stained with 5 Tag-It Violet (BioLegend) following the manufacturer's protocol and then 60000 cells were seeded in a volume of 100 per well in a V-bottom 96 well tissue culture plate. T2 cells were then incubated with 100 of peptide dilution prepared to the desired concentration in complete RPMI for 1 hour at 37. T2 cells were then washed, resuspended in 100 of complete RPMI and transferred to a flat-bottom 96 well tissue culture plate.

Primary T cells were counted and re-suspended in fresh media such that there were 30000 receptor positive cells per 100. This volume was then added to the T2 cells transferred previously.

As controls for the LDH assay additional wells were prepared in triplicate containing only 30000 T cells for each construct, or only 60000 T2 cells. Both with media to the same final volume as the co-cultured cells. Triplicate wells serving as volume correction and media controls were also prepared.

Plates were then spun at 50for 2 minutes and incubated for 8 hours at 37. After this period plates were spun again at 50for 2 minutes and a fraction of supernatant was removed for assessing LDH release. LDH release was assessed using CyQUANT LDH Cytotoxicity Assay Kits (Thermo Fisher) following the manufacturers protocol. EDTA was added to the remaining supernatant (final concentration 2.5) and cells were detached by pipetting.

Cells were stained for CD69 (Clone FN50, dilution 1:200) as well as with pMHC tetramers (dilution 1:500). Stained cells were either analysed immediately or fixed with 1% formaldehyde in PBS and analysed on the following day.

T cells were discriminated from T2 cells by the absence of Tag-It Violet stain. Single T cells were identified on the basis of size and subsequent analysis performed on this population.

Solid-phase plate stimulation. Pierce Streptavidin Coated High Capacity 96 well plates (Thermo Fisher) were washed with PBS and dilutions of biotinylated pMHC in PBS were added to each well in a 50 volume and incubated for 90 minutes at room temperature. Subsequently, plates were washed again with PBS and biotinylated accessory molecules (CD58, ICAM-1, CD86, CD70, 41BBL) were added at a fixed dose of 250 in 50. Plates were again incubated for 90 minutes and then washed with PBS.

T cells were counted, washed in media and 75000 cells in 200 were dispensed per well, Plates were spun for 2 minutes at 50and then incubated for 24 hours at 37. Following this incubation a portion of supernatant was removed and stored for performing ELISAs. EDTA was added to the remaining supernatant (final concentration 2.5) and cells were detached by pipetting. Collected cells were stained for CD45 (Clone HI30, dilution 1:200), CD69 (Clone FN50, dilution 1:200), 4-1BB (Clone 4B4-1, dilution 1:200) and with tetrameric PE-conjugated pMHC. Cells were analysed either immediately or 1 day later, following fixation with 1% formaldehyde in PBS. In a separate assay cells were alternatively stained for CD2 (Clone TS1/8, dilution 1:200), LFA-1 (Clone HI111, dilution 1:200) and tetrameric PE-conjugated pMHC.

Downregulation with Brefeldin A. Cells were prepared as described in the section 'Plate Stimulation', but seeded in a smaller volume of 150 uL/well. Brefeldin A 1000X (BioLegend) was diluted to 4X in sufficient volume for the number of seeded wells and added 50 uL/well after 5 hours of incubation (final concentration 1X, 0.005 mg/ml). Plates were then incubated for the remaining 19 hours of the assay. Harvesting and staining is as in 'Plate Stimulation' with the difference that prior to antibody staining the samples were stained with Zombie nIR viability stain (BioLegend) 1:1000 in 50 uL/well of PBS, and subsequently washed with 200 uL of PBS 1% BSA before proceeding. Samples were not fixed and were run immediately on a flow cytometer. Dead cells were excluded from the subsequent analysis.

Generating U87 knockout cell lines. U87 cells (a kind gift of Vincenzo Cerundolo) were used to generate genetic knockouts for CD58, ICAM1, or both using CRISPR Cas9 RNP transfection. To generate CD58 KO cells, 50,000 U87 cells were seeded in a 24-well plate and transfected the next day using Lipofectamine CRISPRMAX Cas9 Transfection agent (Thermo Fisher), annealed crRNA:tracrRNA (TrueGuide CRISPR758411_CR, `GTCAATGCACAAGTTAGTGT`, Thermo Fisher; A35506 for tracrRNA, Thermo Fisher), and TrueCut Cas9 Protein v2 (Thermo Fisher, A36496) according to manufacturer's instructions. Cells were FAC sorted and this mixed population was used for all experiments. Sorted CD58 KO cells or WT U87 cells were used to generate CD58/ICAM1 double KO cells or ICAM1 KO cells, respectively using the same protocol as above. Specifically, cells were transfected with crRNA:tracrRNA (TrueGuide CRISPR845351_CR, `GCTATTCAAACTGCCCTGAT`, Thermo Fisher) and subsequently FAC sorted. Accutase (Biolegend 423201) was used to dissociate cells before screening or sorting with anti-CD58 (TS2/9, Invitrogen 12-0578-42) or anti-ICAM1 (HA58, Biolegend 353114) to prevent potential digestion of CD58 or ICAM1 by trypsin. All cell lines showed similar expression of HLA-A2 by flow cytometry (clone BB7.2, Biolegend 343306).

APC stimulation (co-culture with U87 cells). 25000 U87 cells were seeded in a tissue culture treated flat-bottom 96 well plate and grown overnight. On the following day the media was removed from these cells and they were incubated with peptides prepared to the appropriate concentration in complete DMEM (DMEM supplemented with 10% v/v FBS, 100 penicillin, 100 streptomycin) for 1 hour at 37.

If blocking antibodies were used then the appropriate amount of T cells were incubated for 30 minutes prior to addition to the U87 cells with either anti-IgG1 Isotype control (BioLegend, Clone MOPC-21), anti-CD58 (BioLegend, Clone TS2/9) or anti-ICAM1 (eBioscience, Clone HA58) at a concentration of 10. Alternatively, both anti-CD58 and anti-ICAM1 together at a concentration of 5 each (total antibody concentration 10).

Peptide containing media was then removed and 50,000 T cells per well were added. The co-culture was then spun for 2 minutes at 50, and incubated for 4 hours at 37. After this period a fraction of supernatant was removed for cytokine ELISAs and stored at -20. EDTA was added to the remaining supernatant (final concentration 2.5) and cells were detached by pipetting.

Cells were stained in PBS 1% BSA for CD45 (Clone HI30, dilution 1:200), CD69 (Clone FN50, dilution 1:200) and 4-1BB (Clone 4B4-1, dilution 1:200) as well as with PE-conjugated tetrameric pMHC (dilution 1:500). Stained cells were either analysed immediately or fixed with 1% formaldehyde in PBS and analysed on the following day.

T cells were discriminated from U87 cells by CD45 staining and/or an assessment of size and complexity. Single T cells were identified on the basis of size and subsequent analysis performed on this population.

Flow cytometry. Tetramers were produced using refolded monomeric biotinylated pMHC and streptavidin-PE (Biolegend) at a 1:4 molar ratio. Streptavidin-PE was added in 10 steps with a 10 minute incubation at room temperature between each addition. 0.05–0.1% sodium azide was added for preservation and tetramers were kept for up to 3 months at 4.

Samples were analysed using a BD LSR Fortessa X-20 (BD Biosciences) or CytoFLEX LX (Beckman Coulter) flow cytometer and data analysis was performed using FlowJo v10 (BD Biosciences).

Electroporation of 868 TCR. 868 TCR alpha and beta chains were amplified using PCR, adding a T7 promoter at the 5′ end. The resulting PCR product was 'cleaned up' using a NucleoSpin Gel and PCR clean-up kit (Macherey-Nagel). Capped and Poly(A) tailed mRNA was produced from this PCR product using a mMESSAGE mMACHINE™ T7 ULTRA Transcription Kit (ThermoFisher). mRNA was collected by lithium chloride precipitation, quality checked by gel electrophoresis and stored in single use aliquots at -80.

For electroporation, T cells are collected and washed 3x with Opti-MEM (Gibco) and resuspended at a concentration of 25e6. 5e6 with 2 per million cells of each of the RNA for the TCR, and chains. Cells were then aliquoted in 200 into an electroporation cuvette (Cuvette Plus 2mm gap BTX). Electroporation is performed using an ECM 830 Square Wave electroporation system (BTX) at 300V for 2. Cells are then transferred to pre-warmed complete RPMI at a density of 1e6. Electroporated cells are used in assays 24 hours later.

Immobilisation Assay. Following a plate stimulation assay, after cells were collected, plates were washed 3 times with PBS 0.05% TWEEN 20 ('PBST') and then stained with anti-HLA-A,B,C (clone W6/32, dilution 1:1000) in PBS for 2 hours at room temperature. Plates were then washed 3x with PBST and stained with secondary goat anti-mouse IgG IRDye 800CW (LI-COR) in PBS for a further 2 hours. Finally plates were washed one more time with PBST and then imaged using a LICOR Odyssey Sa (LI-COR). Integrated intensity per well is reported.

ELISAs. Invitrogen Uncoated ELISA kits for IFN (Thermo Fisher) were used following the manufacturer's protocol. Supernatants were either used immediately for ELISAs post-harvesting or stored at -20 for up-to 2 weeks. Supernatants were diluted using an empirically determined ratio before use in an ELISA so that quantities of assessed cytokines fell within the linear range of the kits.

Surface Plasmon Resonance. D52N–pMHC interactions were analysed on a Biacore T200 instrument (GE Healthcare Life Sciences) at 37°C and a flow rate of 30 l/min. Running buffer was HBS-EP (10 mM HEPES pH 7.4, 150 mM NaCl, 3 mM EDTA, 0.005% v/v Tween-20). Streptavidin was coupled to CM5 sensor chips using an amino coupling kit (GE Healthcare Life Sciences) to near saturation, typically 10,000–12,000 response units (RU). Biotinylated pMHCs (47 kDa) were injected into the experimental flow cells (FCs) for different lengths of time to produce desired immobilisation levels (300–1000 RU). FC1 and FC3 were used as reference FCs for FC2 and FC4, respectively. Biotinylated ECD of CD58 (24 kDa + ∼25 kDa glycosylation) was immobilised in the reference FCs at levels matching those of pMHCs. Excess streptavidin was blocked with two 40 s (D52N STAR) or 60 (D52N scFv) injections of 250 M biotin (Avidity). Before injections of purified D52N, the chip surface was conditioned with eight injections of the running buffer. Dilution series of D52N were injected simultaneously in all FCs starting from the lowest concentration, which was injected again after the highest concentration to confirm stability of pMHC on the chip surface. The duration of injections (20 or 180 s) was the same for conditioning and D52N injections. After every 2 or 3 D52N injections, buffer was injected to generate data for double referencing. In addition to subtracting the signal from the reference FC (single referencing), all D52N binding data were double referenced versus the average of the closest buffer injections before and after D52N injection to correct for small differences in signal between flow cells. D52N binding versus D52N concentration was fitted with the following model: $B = B_{max} \cdot \frac{[D52N]}{K_D + [D52N]}$, where B is the response (binding) and B_{max} is the maximal binding.

Sequences. D52N scFvs with the following sequence were produced by Absolute Antibody Ltd.

D52N scFv:

```
EVQLLESGGGLVQPGGSLRLSCAASGFTFSTYQMSWVRQAPGKGLEW
VSGIVSSGGSTAYADSVKGRFTISRDNSKNTLYLQMNSLRAEDTAVY
YCAGELLPYYGMDVWGQGTTVTVSSAKTTPKLEEGEFSEARVQSELT
QPRSVSGSPGQSVTISCTGTERDVGGYNYVSWYQQHPGKAPKLIIHN
VIERSSGVPDRFSGSKSGNTASLTISGLQAEDEADYYCWSFAGGYYV
FGTGTDVTVLG
```

The D52N-IgG1 CARs contain a 'HNG spacer sequence' derived from the IgG1 hinge region, described in (4), and spliced with a spacer region from the CH2-CH3 regions of IgG1 as described in (5).

HNG Spacer:

```
DPAEPKSPDKTHTCPPCP
```

The 1G4 TCR and chains are joined by a P2A linker peptide with an additional spacer and furin cleavage site, as described in (6). The sequence is given below.

Furin-P2A:

```
GSRAKRSGSGATNFSLLKQAGDVEENPGP
```

Independent experiments and data analysis. To produce independent measurements of EC_{50} (individual data points in figure panels) for a given antigen receptor, we produced a new batch of lentivirus which was used to transduce T cells isolated from a new leukocyte cone, which is provided by the National Health Service (NHS) in the UK and is obtained from human blood donors.

In each independent experiment, we included the TCR and one or more CARs to be tested and used pMHC antigen tetramers to evaluate the percent of T cells expressing each antigen receptor (Fig. SS2C, transduction efficiency) and the surface level (gMFI of T cells expressing the antigen receptor) for each antigen receptor relative to the TCR (Fig. SS2D). Although we observed variations in the transduction efficiency, the surface level of each antigen receptor was always at the same level or higher compared to the TCR.

As a result of differences in the transduction efficiency, we observed differences in the maximum number of T cells that could upregulate CD69 or 4-1BB across independent experiments for the same antigen receptor or across different antigen receptors

(see y-axis in Fig. 2B,C for example). These differences reflect the percent of T cells that express the antigen receptor and can therefore respond to the presented antigen. Importantly, our study focused on measuring antigen sensitivity (), which is defined as the concentration of antigen required to elicit half-maximal response. Therefore, variations in the maximum number of T cells able to respond are taken into account when measuring an .

Statistical analysis was performed using Prism (GraphPad Software) or Excel (Microsoft). Curve fitting was performed using the robust nonlinear regression function in Prism or MATLAB (MathWorks) and the EC_{50} extracted from the fitted curves. Data was excluded from analysis if the computed fit was reported as 'ambiguous' in Prism, if the fit did not converge in 1000 iterations, or if the computed EC_{50} was outside of the tested ligand concentration.

All data is included in the manuscript figures.

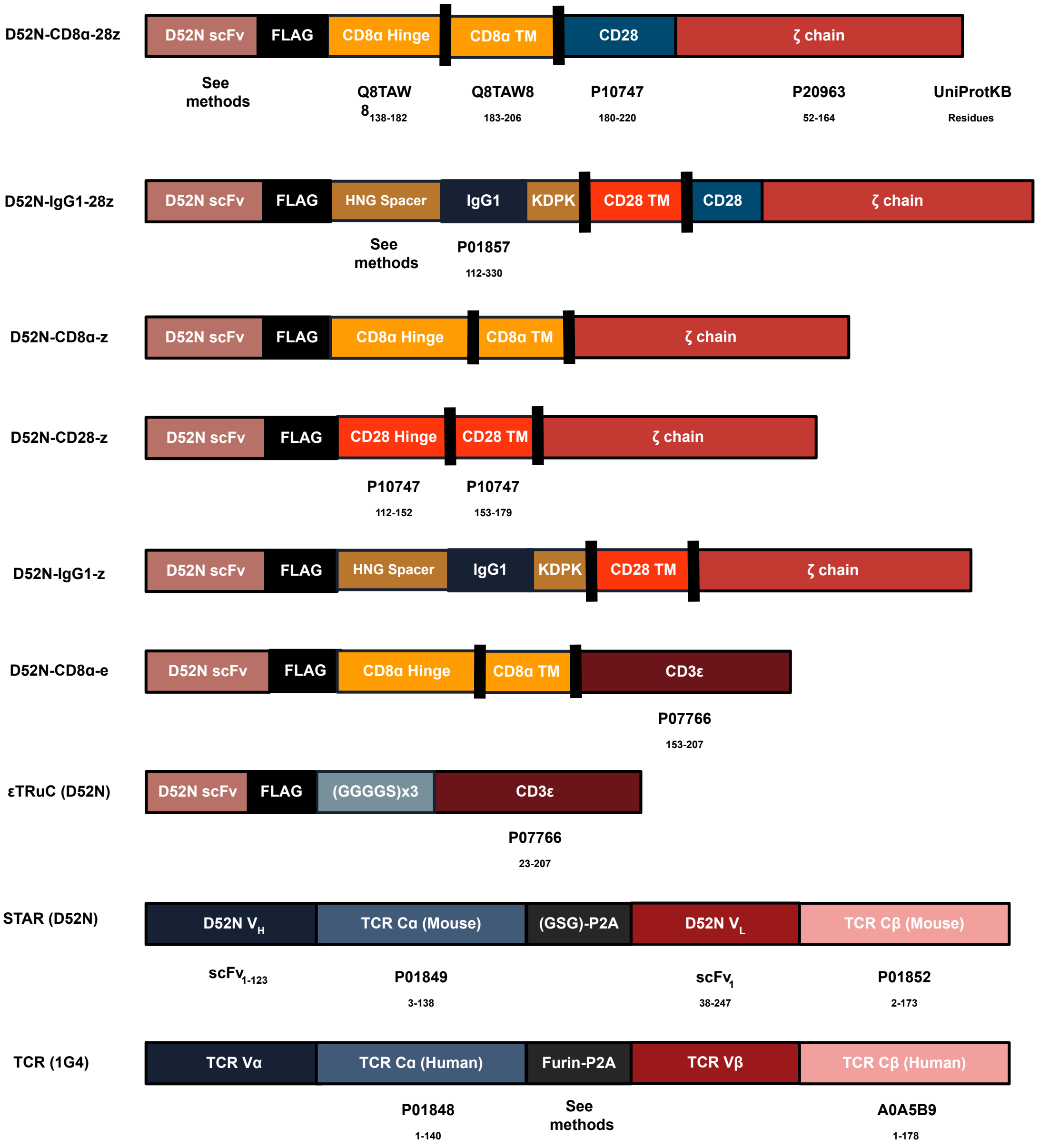

Fig. S1. Schematic of antigen receptor architectures used in the study.

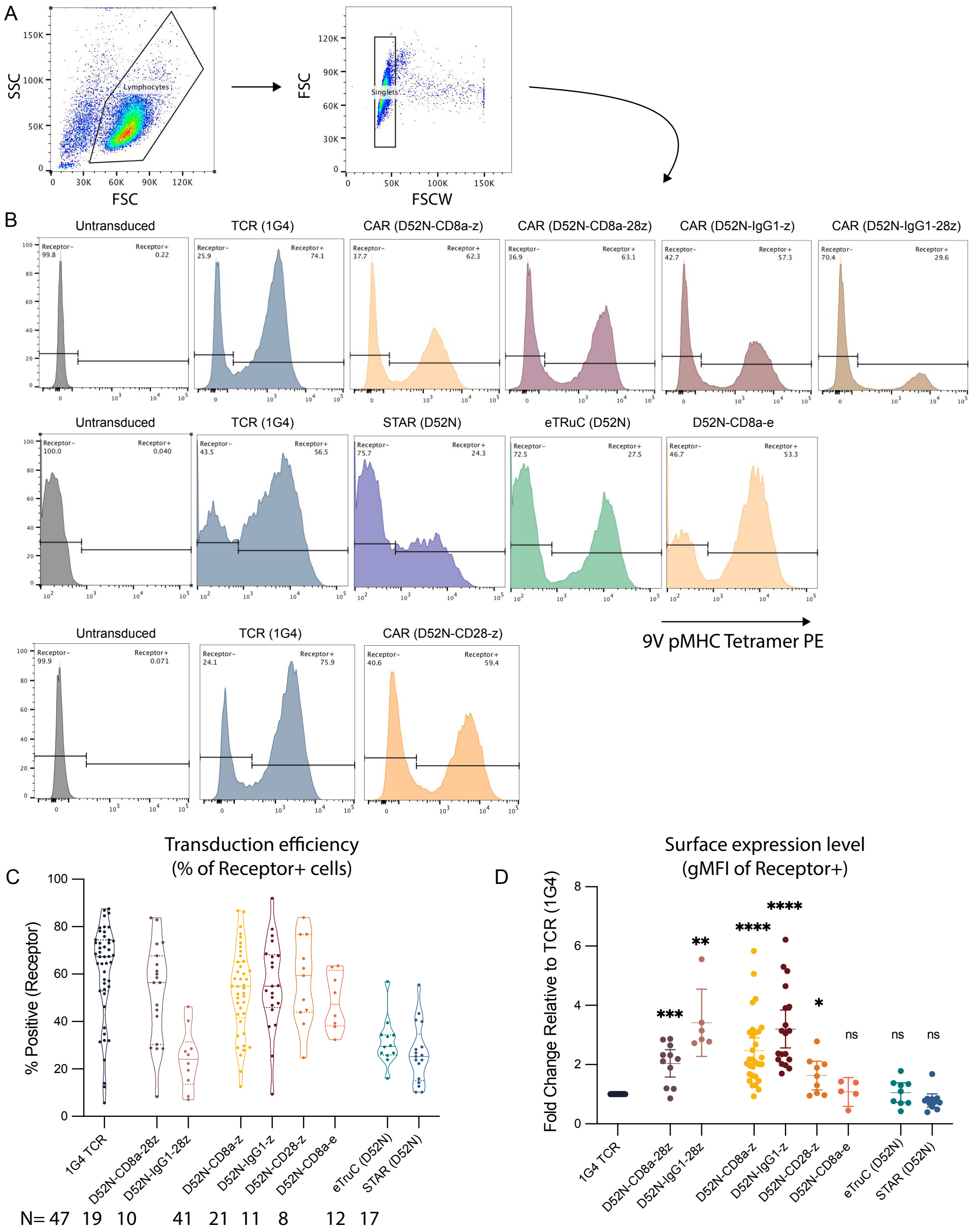

Fig. S2. Surface expression of chimeric receptors was similar or higher compared to the TCR. **(A)** Gating strategy to identify single lymphocytes. **(B)** Representative flow cytometry histograms showing surface expression of the indicated surface receptor using fluorescent 9V pMHC tetramers. Untransduced T cells are used to determine the negative gate. Each row is an independent experiment and each column is the indicated antigen receptor with the TCR being included in all experiments performed in the study. **(C)** The percent of T cells expressing the indicated receptor (i.e. within receptor positive gate). **(D)** The fold-change in the surface expression of each chimeric antigen receptor relative to the TCR determined by the gMFI of T cells in the receptor positive gate. The surface expression of each antigen receptor was determined for every experiment carried out in the study and is shown in aggregate in panel C and D. Individual data points for each antigen receptors represents an independent experiment (N is shown below the labels in panel C), which is generated by producing lentivirus and transducing a new sample of primary human T cells (see Methods). The TCR contains the largest number of experiments because it was included in every experiment. A one-sample t-test is used to compare each chimeric receptor to the expression of the TCR (1.0) on log-transformed values. Abbreviations: * = p-value≤0.05, ** = p-value≤0.01, *** = p-value≤0.001, **** = p-value≤0.0001.

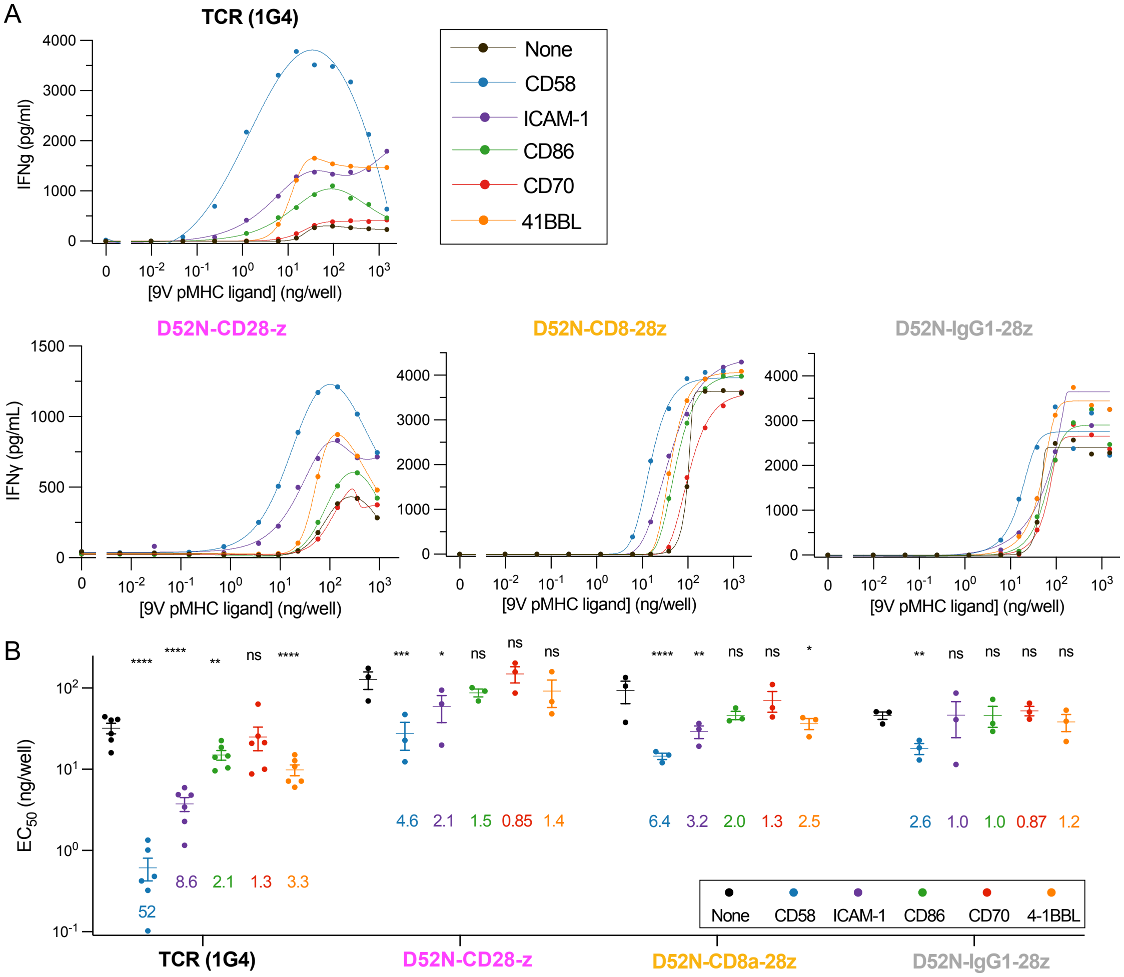

Fig. S3. Extended data for Figure 2 confirming that CARs are inefficient at exploiting adhesion receptors when measuring IFNγ production. (A) Representative dose-responses using T cells expressing the indicated antigen receptor stimulated by a titration of purified pMHC alone ('None') or in combination with a fixed concentration of the indicated purified accessory receptor ligand (colours). The supernatant concentration of IFNγ was determined after 24 hours using ELISA. Each dose-response curve was fitted to obtain the and E_{max} values. **(B)** The values for the indicated antigen receptor and purified ligand. The coloured numbers indicate the fold-change in induced by the addition of the indicated accessory receptor ligand relative to pMHC alone ('None'). The values for each ligand are compared to the 'None' condition using a paired t-test on log-transformed data. **(C)** The fold-change in E_{max} relative to pMHC alone ('None') for each antigen receptor and purified ligand. The fold-change is compared using a one-sample t-test to a hypothetical value of 0 on log-transformed data. Abbreviations: * = p-value$\leq$0.05, ** = p-value$\leq$0.01, *** = p-value$\leq$0.001, **** = p-value$\leq$0.0001.

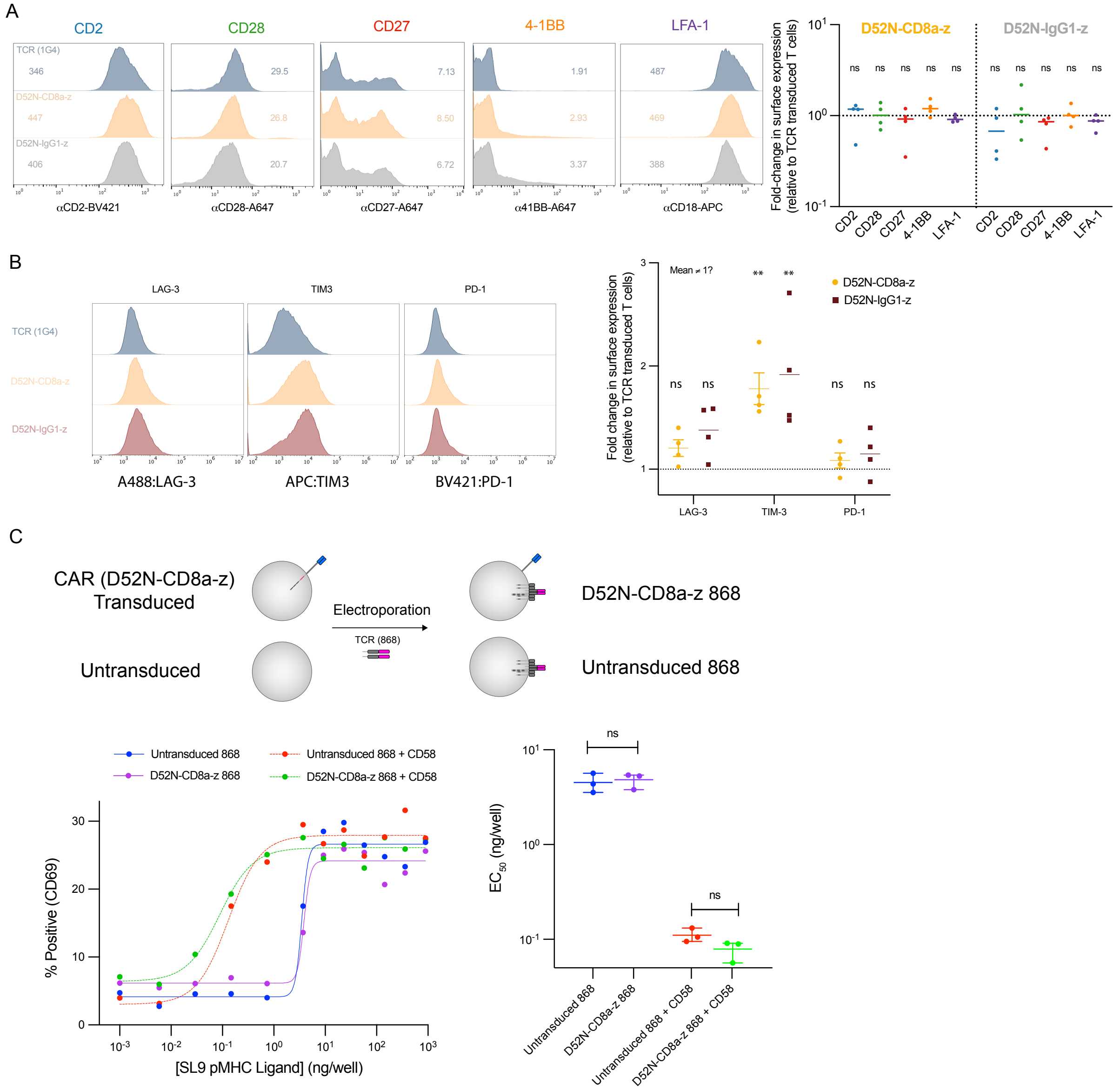

Fig. S4. The CAR antigen sensitivity defect is not a result of exhaustion induced by tonic signalling. (A) Surface expression of the indicated co-stimulation receptors on T cells transduced with the TCR or the indicated CAR. Representative flow cytometry histograms (top) and fold-change across independent experiments (N=4, bottom). **(B)** Surface expression of the indicated co-inhibitory receptors on T cells transduced with the TCR or with the indicated CARs. Representative flow cytometry histograms (left) and fold-change across independent experiments (N=4, right). **(C)** CAR transduced or untransduced T cells are electroporated with the 868 TCR specific to the SL9 peptide antigen before being stimulated by purified SL9 pMHC with or without CD58. Representative dose-response (bottom left) and EC_{50} values across independent experiments (N=3, bottom right). A one-sample t-test is used to obtain a p-value for the null hypothesis that the indicated surface receptor expression differs from 1.0 on log-transformed values (panel A and panel B, right) and a two-sample t-test is used to compare log-transformed (panel C, bottom right). Abbreviations: * = p-value$\leq$0.05, ** = p-value$\leq$0.01, *** = p-value$\leq$0.001, **** = p-value$\leq$0.0001.

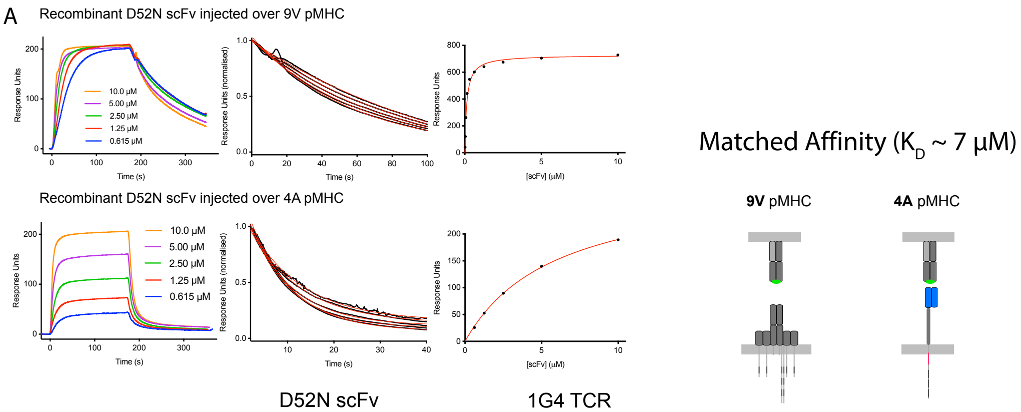

	D52N scFv				1G4 TCR
pMHC	k_{on} ($\mu M^{-1}s^{-1}$)	k_{off} (s^{-1})	K_D (μM)	n	K_D (μM)
9V (SLLMWITQV)	0.087	0.013±0.001	0.15±0.03	3	**7.2±0.5**
4A (SLLAWITQV)	0.021	0.15±0.07	**7.0±1.4**	2	299±49

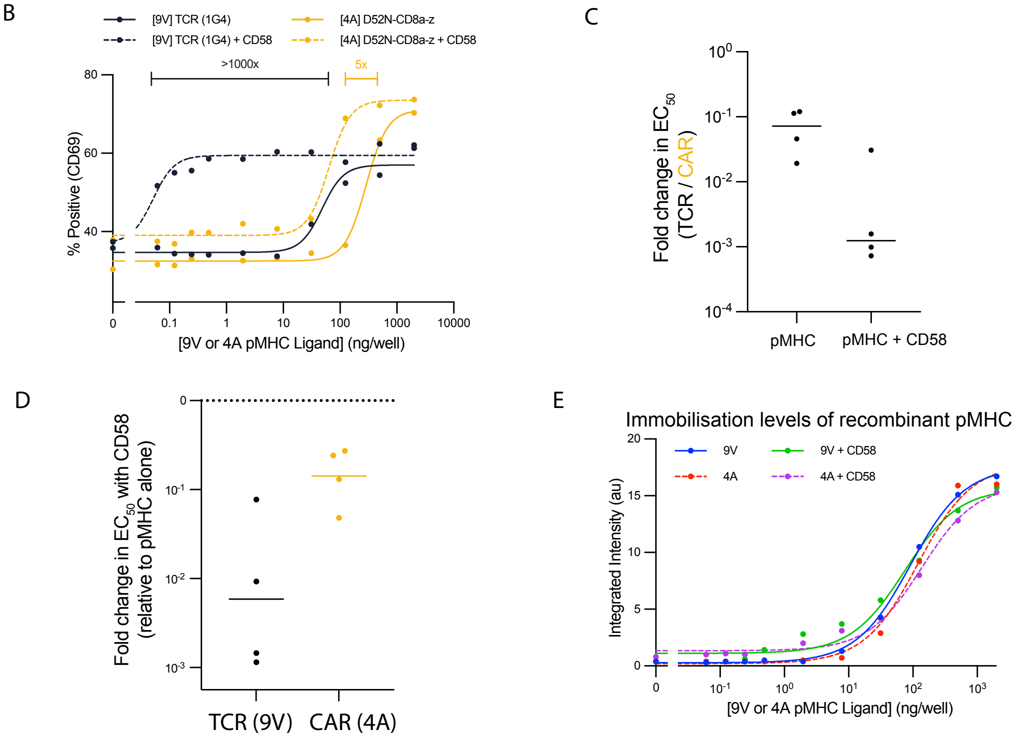

Fig. S5. Matching the antigen affinity of the TCR and CAR increases the antigen sensitivity defect of CARs. (A) Binding between purified D52N scFv and the 9V (top) or 4A (bottom) pMHCs measured by surface plasmon resonance (SPR) showing the full sensogram (left), dissociation phase (middle) and steady state binding response (right). The kinetic and equilibrium are obtained by fitting the dissociation phase and steady-state binding response, respectively (red line is model fit). The kinetic is derived from and . The values for the TCR are obtained from previous work (2). **(B)** Representative dose-response for the TCR recognising the 9V pMHC and for the CAR recognising the 4A pMHC with (dashed line) or without (solid line) purified CD58 (250 ng/well). **C-D** Fold-change in between (C) the TCR recognising 9V and the CAR recognising 4A and (D) induced by the addition of CD58 for the indicated pMHC and antigen receptor across independent experiments (N=4). **(E)** Levels of presented pMHC for each condition as detected by the conformationally sensitive W6/32 antibody.

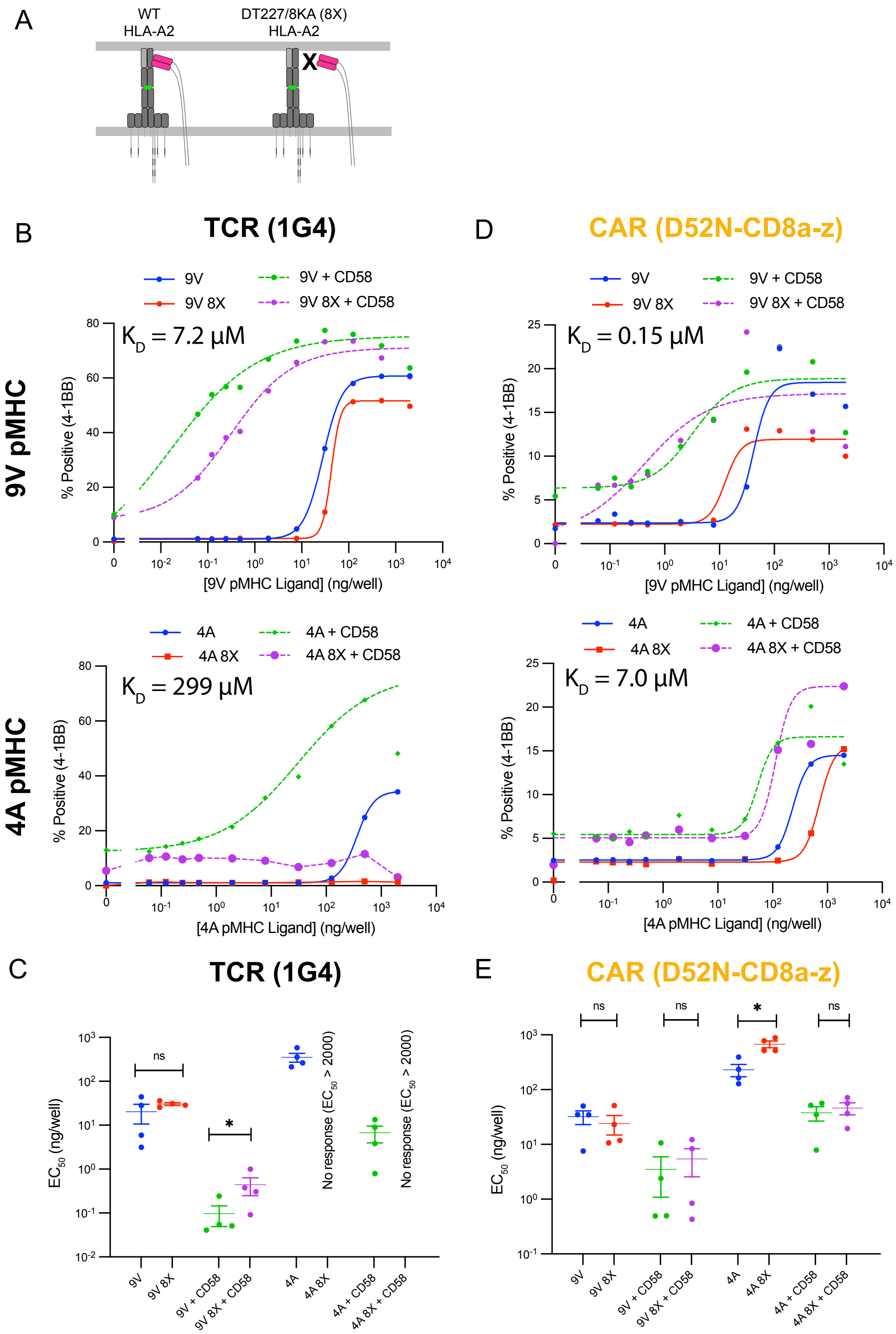

Fig. S6. The CAR antigen sensitivity defect is independent of the CD8 co-receptor. (A) The DT227/8KA mutations in the HLA-A2 heavy-chain prevent binding by the CD8 co-receptor (referred to as 8X). **(B-E)** Representative dose-response curves (B,D) and summary measures across independent experiments (N=4) (C,E) for the 9V and 4A pMHC variants with or without CD58 for the TCR (B,C) and the CAR (D,E). A t-test is used to compare the values on log-transformed data. Abbreviations: * = p-value$\leq$0.05, ** = p-value$\leq$0.01, *** = p-value$\leq$0.001, **** = p-value$\leq$0.0001.

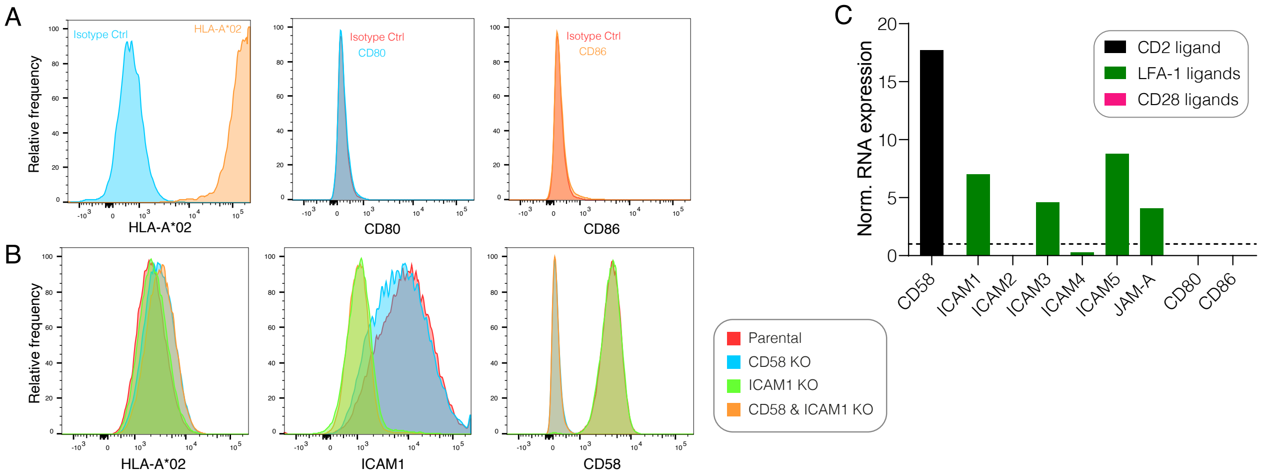

Fig. S7. Expression of accessory receptor ligands on the U87 glioblastoma cell line. (A) Expression of HLA-A*02 (left), CD80 (middle), and CD86 (right) on parental U87 cells. **(B)** Expression of HLA-A*02 (left), ICAM-1 (middle), and CD58 (right) on the parental U87 cells or the indicated knockout cell lines. **(C)** Expression of the indicated molecule by RNA as reported in the Human Cell Atlas (7).

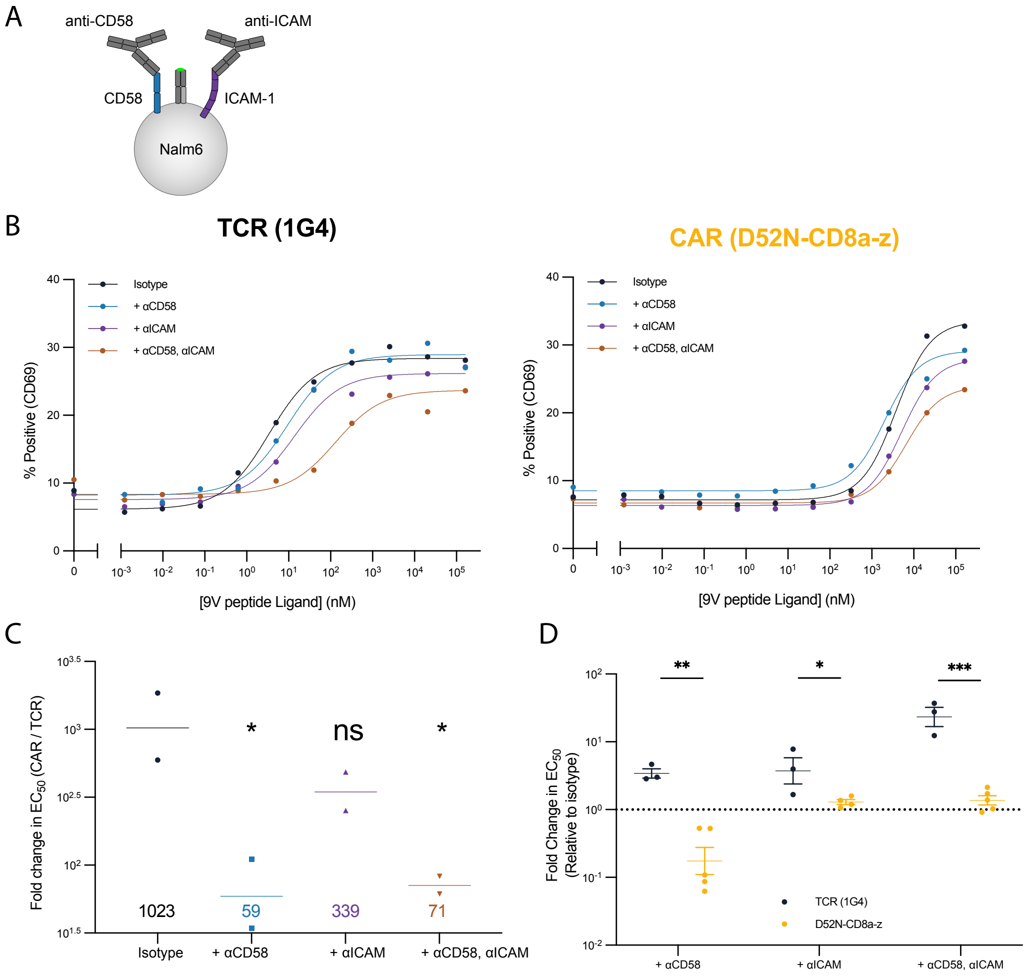

Fig. S8. Abrogating the CD2 and LFA-1 adhesion interaction disproportionately impact the antigen sensitivity of the TCR compared to the CAR when recognising antigen on the Nalm6 target cell line. **(A)** Schematic of CD58 and ICAM-1 blocking experiment on the HLA-A2+ Nalm6 leukemia cell line. **(B)** Representative dose-response curves for the indicated blocking conditions for the TCR (left) and CAR (right). **(C)** Fold-change in between the CAR and TCR for the indicated condition. **(D)** Fold-change in relative to the isotype for the indicated condition. The fold-change between the TCR and CAR is compared using a two-sample t-test to the isotype (panel C) or directly between the TCR and CAR (panel D) on log-transformed values. Abbreviations: * = p-value $\leq$0.05, ** = p-value $\leq$0.01, *** = p-value $\leq$0.001.

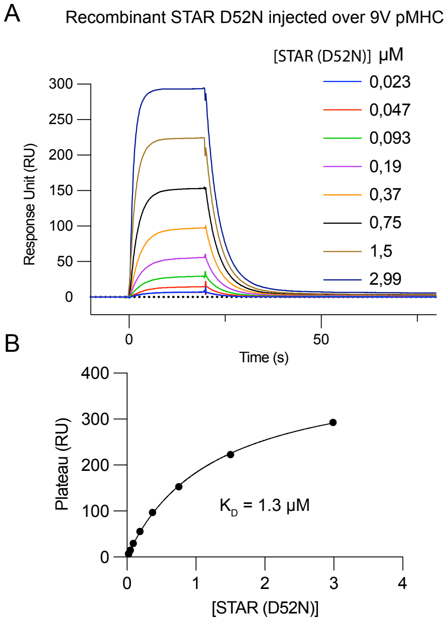

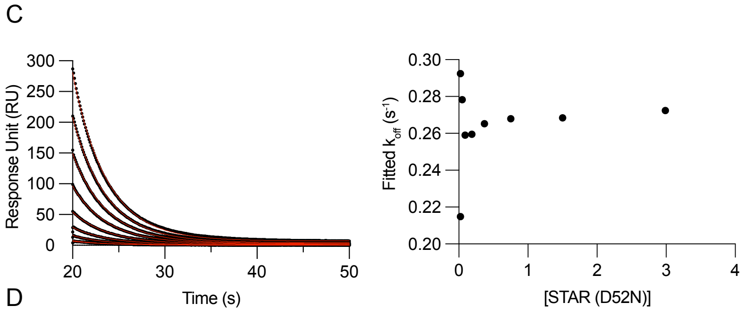

D

STAR (D52N)

pMHC	k_{on} ($\mu M^{-1}s^{-1}$)	k_{off} (s^{-1})	K_D (µM)	n
9V (SLLMWITQV)	0.20±0.02	0.25±0.001	1.3±0.1	4

Fig. S9. The binding affinity and kinetics between recombinant STAR (D52N) and 9V pMHC. (A) Representative binding between purified D52N STAR at the indicated concentration injected over a surface with immbolised 9V pMHC measured by surface plasmon resonance (SPR) showing the full sensogram. **(B)** The equilibrium is obtained by fitting the steady-state response. **(C)** The kinetic for each experiment is obtained by fitting the dissociation phase (left) and averaging the values for different concentrations (right). **(D)** Summary of binding constants with the kinetic determined for each experiment using and .

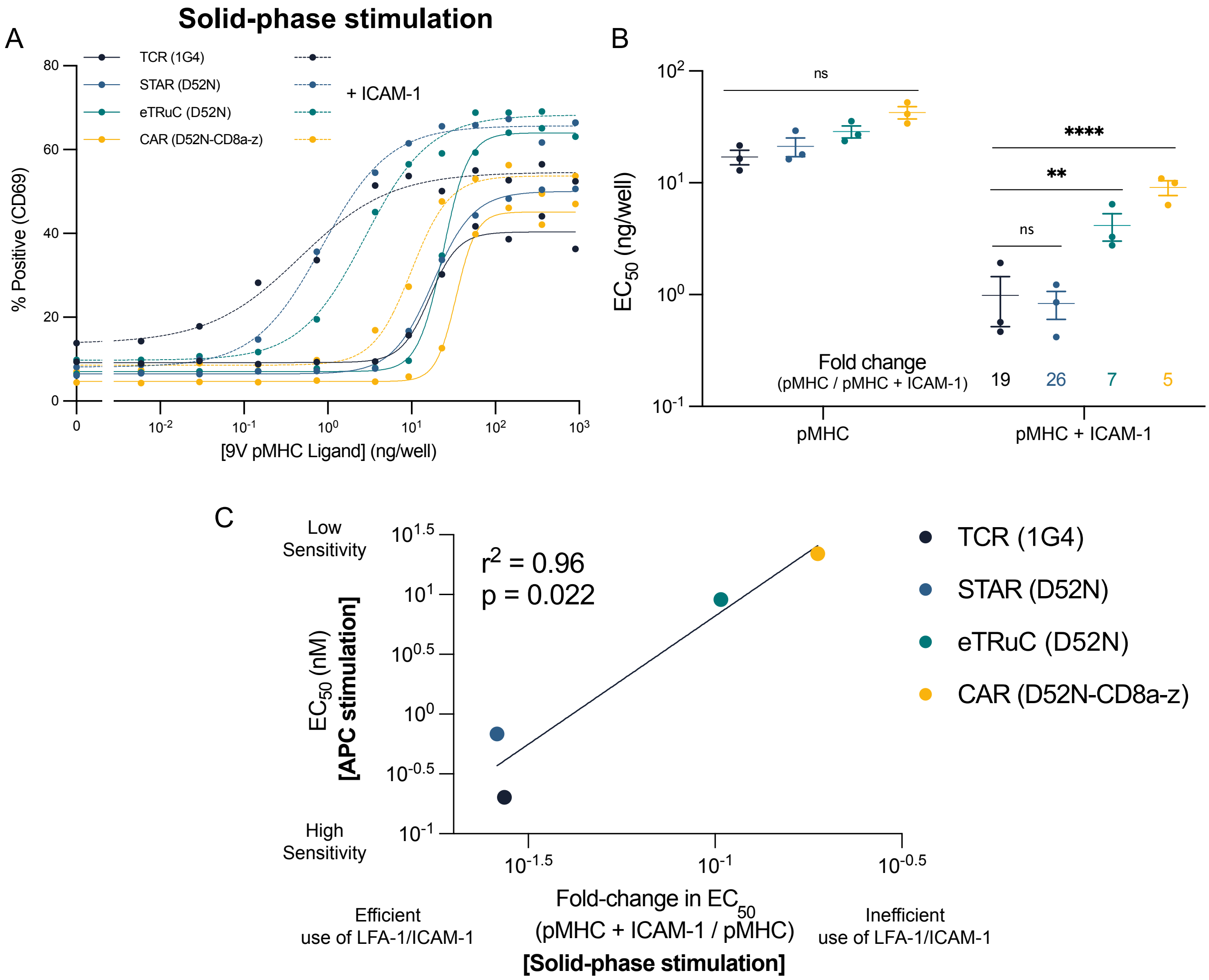

Fig. S10. The ability of TCR-like chimeric antigen receptors to recapitulate the sensitivity of the TCR depends on the efficiency with which they are able to exploit the LFA-1 adhesion interaction. (A) T cells expressing the indicated antigen receptor were stimulated by a titration of purified pMHC alone (solid lines) or in combination with a fixed concentration of purified ICAM-1 (dashed lines). **(B)** Fitted values from two independent experiments. **(C)** The averaged values for CD69 upregulation from the APC stimulation assay (from Fig. 4E) are plotted over the averaged fold-change in for CD69 induced by the addition of ICAM-1 from the solid-phase stimulation assay (from panel B). The values are compared using a one-way ANOVA on log-transformed values (B). Abbreviations: *** = p-value$\leq$0.001, **** = p-value$\leq$0.0001.

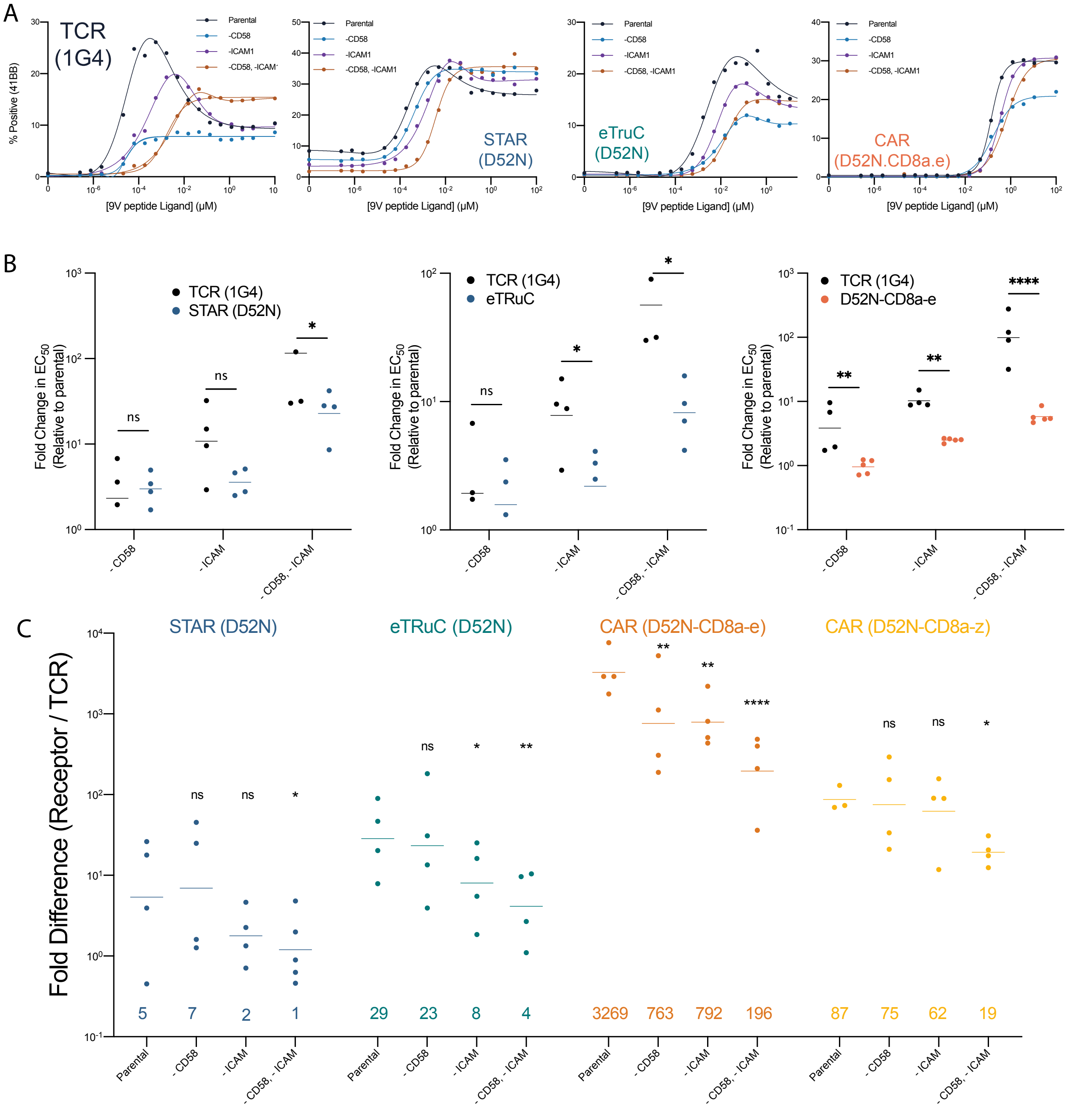

Fig. S11. Abrogating the CD2 and LFA-1 adhesion interaction disproportionately impact the antigen sensitivity of the TCR compared to the eTruc and the ε-chain CAR. (A) Representative dose-response curves for the indicated U87 knockout target cell line for the indicated antigen receptor. **(C)** Fold-change in relative the parental U87 target line for the STAR (left), eTruC (middle), and ε-chain CAR (right). The TCR was included in each experiment. **(C)** Fold-change in between the TCR and the indicated antigen receptor (top labels) for the indicated U87 target cell (bottom labels). The EC50 values are shown fro at least 3 independent experiments. The fold-change is compared using a two-sample t-test on log-transformed data. Abbreviations: * = p-value$\leq$0.05, ** = p-value$\leq$0.01, *** = p-value$\leq$0.001, **** = p-value$\leq$0.0001.

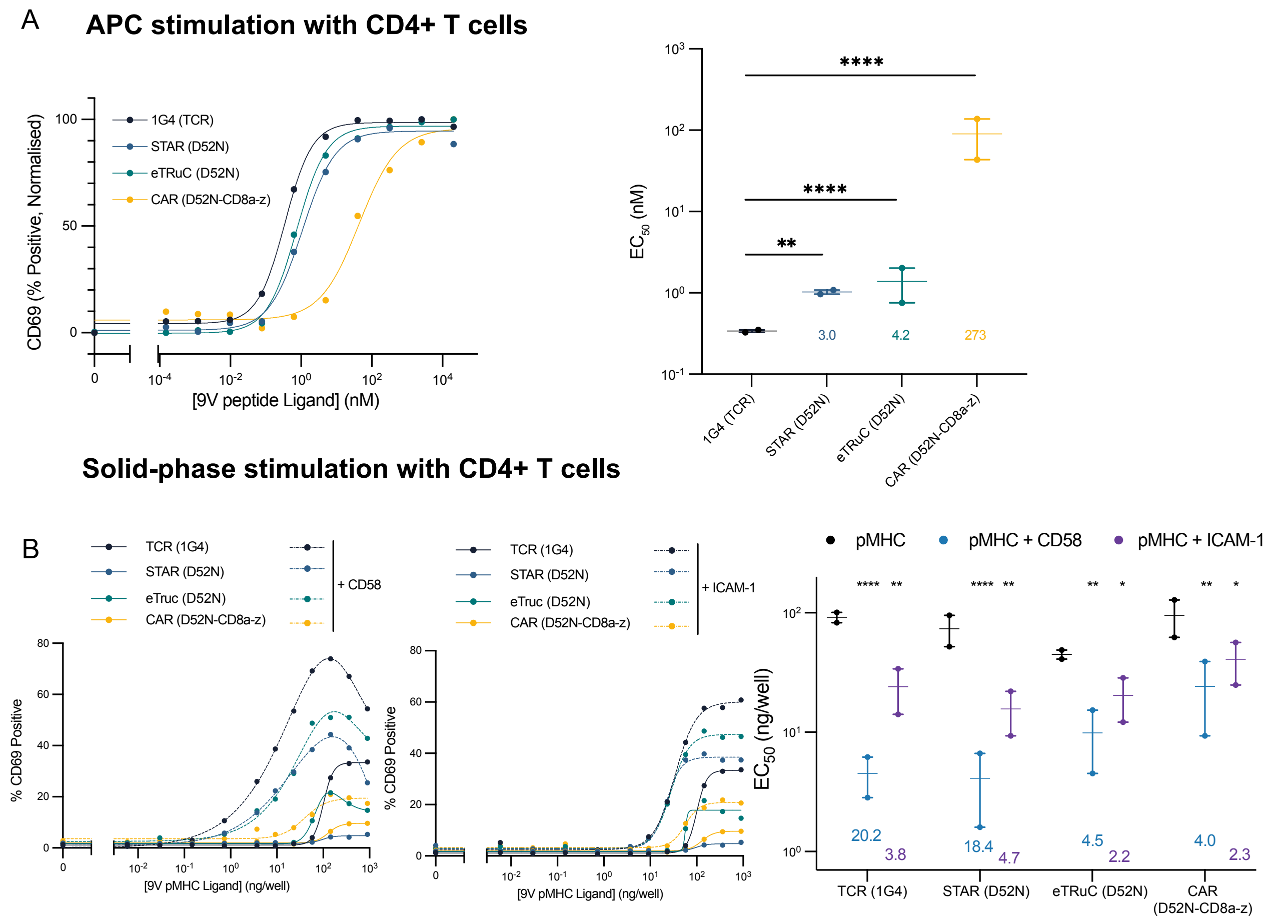

Fig. S12. CD4^{+} CAR-T cells exhibit reduced antigen sensitivity and inefficient exploitation of CD2 and LFA-1 compared to the TCR. (A) CD4+ T cells expressing the indicated antigen receptor were stimulated by T2 cells pulsed with the indicated peptide concentration. Representative dose-response (left) and values (right) from N=2 independent samples. **(B)** CD4+ T cells expressing the indicated antigen receptor were stimulated by a titration of purified pMHC alone or in combination with a fixed concentration of purified CD58 or ICAM-1. Representative dose-response (left) and values (right) from N=2 independent samples. The values are compared using a one-way ANOVA on log-transformed values. Abbreviations: * = p-value$\leq$0.05, ** = p-value$\leq$0.01, **** = p-value$\leq$0.0001.

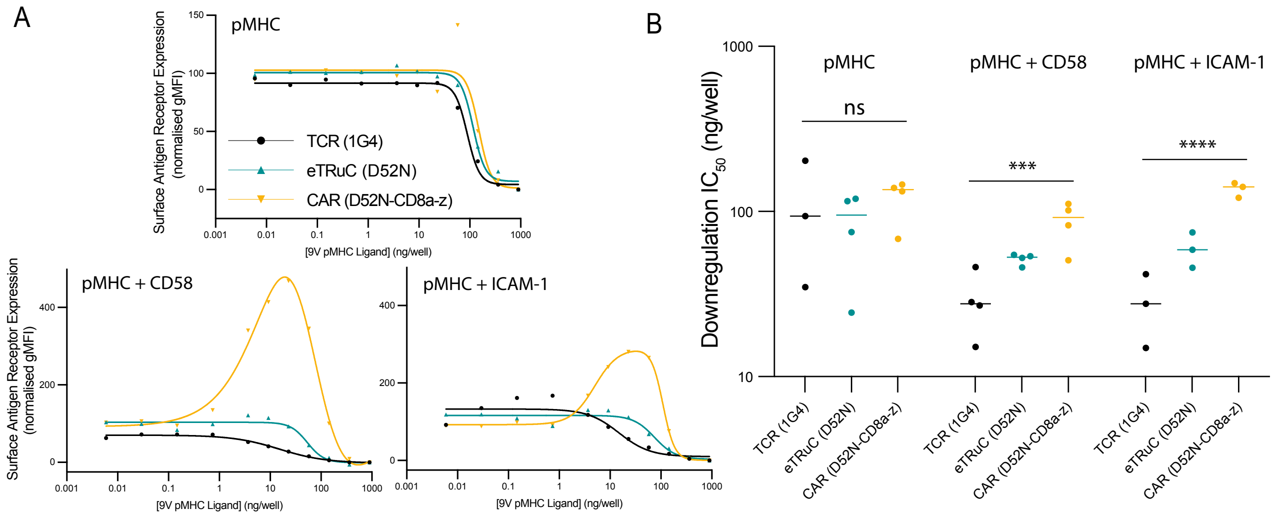

Fig. S13. Adhesion receptors more efficiently enhance antigen-dependent downregulation of the TCR compared with the eTruC and CAR. (A-B) Effect of pMHC with or without CD58 or ICAM-1 on antigen receptor expression, as determined by pMHC tetramers. (A) Representative curves and (B) fitted IC_{50} values from at least 3 independent experiments. All data is normalised to surface expression without pMHC. The IC_{50} is determined to the right of the bell-shapes observed for the CAR with CD58 and ICAM-1. This experiment was performed without the addition of Brefelding A. All comparisons are made using a one-way ANOVA on log-transformed values. Abbreviations: *** = p-value$\leq$0.001, **** = p-value$\leq$0.0001.

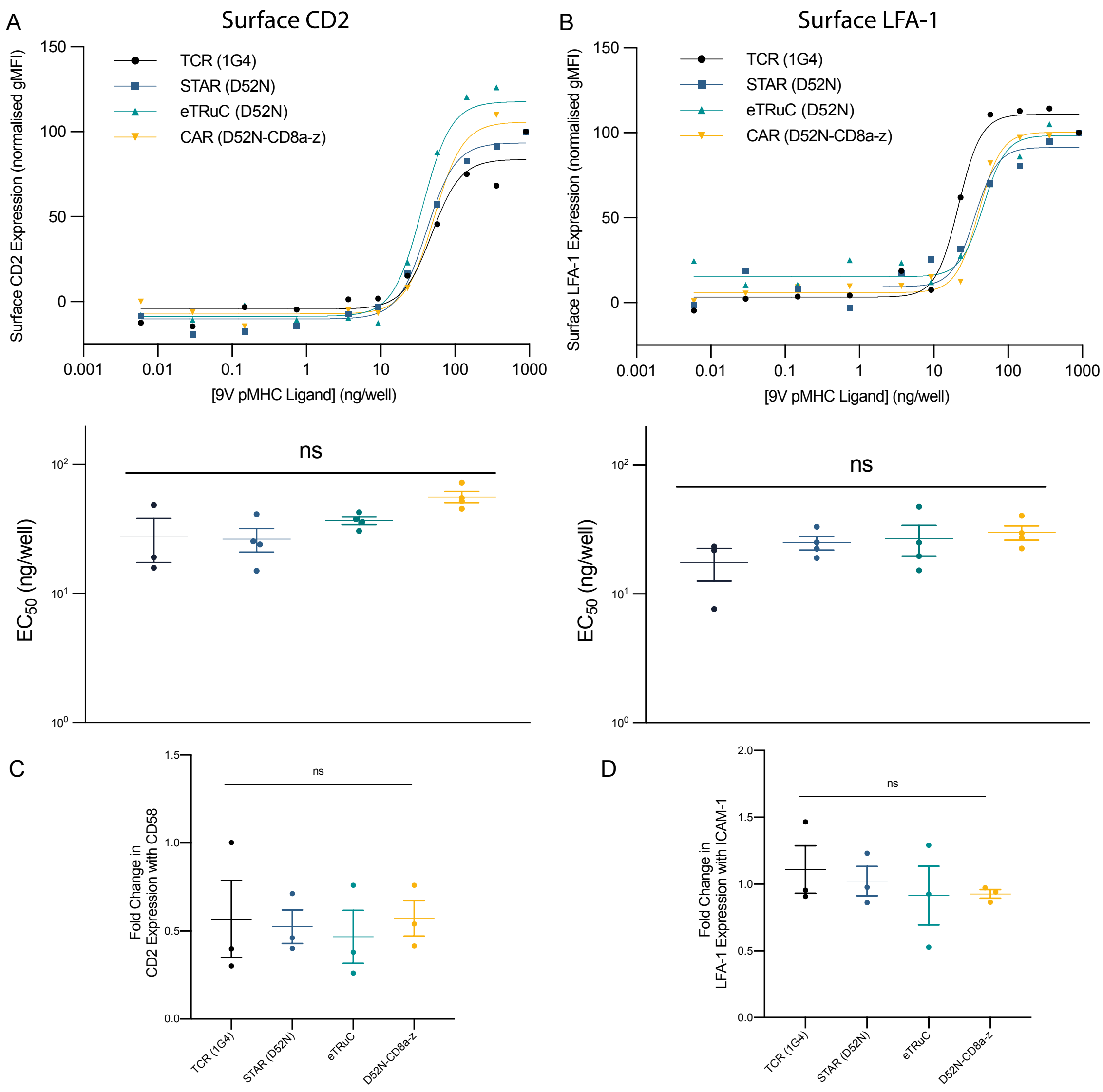

Fig. S14. Up-regulation of surface CD2 and LFA-1 is similar for different antigen receptors. (A) Surface CD2 or **(B)** surface LFA-1 on T cells expressing the indicated antigen receptors stimulated in the solid-phase stimulation with antigen alone. The LFA-1 antibody clone used in the present work recognises the inactive conformation of LFA-1. Surface expression is normalised to the condition without antigen. The values are compared using a one-way ANOVA on log-transformed values. **(C,D)** The fold-change in (C) surface CD2 or (D) LFA-1 for T cells transduced with the indicated antigen receptor stimulated in the solid-phase stimulation with CD58 or ICAM-1, respectively in the absence of antigen.

References

1. Achour A, et al. (1999) Murine class i major histocompatibility complex h–2dd: expression, refolding and crystallization. *Acta Crystallographica Section D: Biological Crystallography* 55:260–262.
2. Pettmann J, et al. (2021) The discriminatory power of the t cell receptor. *eLife* 10:1–42.
3. Boulter JM, et al. (2003) Stable, soluble t-cell receptor molecules for crystallization and therapeutics. *Protein engineering* 16:707–711.
4. Patel A (2017) Ph.D. thesis (UCL (University College London)).
5. Watanabe N, et al. (2016) Fine-tuning the car spacer improves t-cell potency. *Oncoimmunology* 5:e1253656.
6. Yang S, et al. (2008) Development of optimal bicistronic lentiviral vectors facilitates high-level tcr gene expression and robust tumor cell recognition. *Gene therapy* 15:1411–1423.
7. Uhlén M, et al. (2015) Tissue-based map of the human proteome. *Science* 347.
